# Reaction Mechanism and Performance of Innovative 2D Germanane‐Silicane Alloys: Si*_x_* Ge_1−_ *_x_* H Electrodes in Lithium‐Ion Batteries

**DOI:** 10.1002/advs.202308955

**Published:** 2024-04-22

**Authors:** Shuangying Wei, Tomáš Hartman, Stefanos Mourdikoudis, Xueting Liu, Gang Wang, Evgeniya Kovalska, Bing Wu, Jalal Azadmanjiri, Ruizhi Yu, Levna Chacko, Lukas Dekanovsky, Filipa M. Oliveira, Min Li, Jan Luxa, Saeed Jamali Ashtiani, Jincang Su, Zdeněk Sofer

**Affiliations:** ^1^ Department of Inorganic Chemistry University of Chemistry and Technology Prague Technická 5 Prague 6 16628 Czech Republic; ^2^ School of Materials Science and Engineering Xiangtan University Xiangtan 411105 China; ^3^ Department of Engineering Faculty of Environment, Science and Economy University of Exeter Exeter EX4 4PY United Kingdom; ^4^ Institute of Micro/Nano Materials and Devices Ningbo University of Technology Ningbo 315211 China; ^5^ School of Physics Xi'an Jiaotong University Xi'an 710049 China; ^6^ Department of Physical Chemistry University of Chemistry and Technology Prague Technická 5 Prague 6 16628 Czech Republic

**Keywords:** 2D materials, DTF calculation, lithium‐ion batteries, reaction mechanisms, silicane‐germanane alloys

## Abstract

The adjustable structures and remarkable physicochemical properties of 2D monoelemental materials, such as silicene and germanene, have attracted significant attention in recent years. They can be transformed into silicane (SiH) and germanane (GeH) through covalent functionalization via hydrogen atom termination. However, synthesizing these materials with a scalable and low‐cost fabrication process to achieve high‐quality 2D SiH and GeH poses challenges. Herein, groundbreaking 2D SiH and GeH materials with varying compositions, specifically Si_0.25_Ge_0.75_H, Si_0.50_Ge_0.50_H, and Si_0.75_Ge_0.25_H, are prepared through a simple and efficient chemical exfoliation of their Zintl phases. These 2D materials offer significant advantages, including their large surface area, high mechanical flexibility, rapid electron mobility, and defect‐rich loose‐layered structures. Among these compositions, the Si_0.50_Ge_0.50_H electrode demonstrates the highest discharge capacity, reaching up to 1059 mAh g^−1^ after 60 cycles at a current density of 75 mA g^−1^. A comprehensive ex‐situ electrochemical analysis is conducted to investigate the reaction mechanisms of lithiation/delithiation in Si_0.50_Ge_0.50_H. Subsequently, an initial assessment of the *c*‐Li_15_(Si*_x_* Ge_1‐_ *_x_*)_4_ phase after lithiation and the *a*‐Si_0.50_Ge_0.50_ phase after delithiation is presented. Hence, this study contributes crucial insights into the (de)lithiation reaction mechanisms within germanane‐silicane alloys. Such understanding is pivotal for mastering promising materials that amalgamate the finest properties of silicon and germanium.

## Graphical abstract

2D materials with compositions Si_0.25_Ge_0.75_H, Si_0.50_Ge_0.50_H and Si_0.75_Ge_0.25_H, are synthesized a simple and efficient chemical exfoliation of their Zintl phases. Among these, the Si_0.50_Ge_0.50_H electrode displays the most superior performance, boosting a discharge capacity of 1059 mAh g^−1^ following 60 cycles at a current density of 75 mA g^−1^. A comprehensive ex‐situ electrochemical analysis is performed to study the reaction mechanisms of lithiation/delithiation.

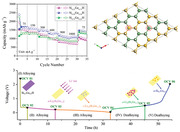

## 1 Introduction

Silicon (Si) and germanium (Ge) have emerged as auspicious anode candidates for lithium‐ion batteries (LIBs), with the potential to supersede graphite. This is attributed to their remarkable theoretical capacities (≈4200 mAh g^−1^ and ≈1600 mAh g^−1^ corresponding to Li_22_Si_5_ and Li_22_Ge_5_, respectively), favorable operating potentials (< 0.31 V vs. Li^+^/Li and < 0.5 V vs. Li^+^/Li), and environmentally friendly characteristics.^[^ ^1^, ^2^ ^]^ Si has received greater attention, while Ge is often overlooked due to its higher cost and lower specific capacity.^[^ ^3^ ^]^ Nevertheless, Ge, boasting a remarkable electrical conductivity of 2.12 S m^−1^,^[^ ^4^ ^]^ along with a fast lithium diffusivity of 1.2 × 10^–11^ cm^2^ s^−1^,^[^ ^5^ ^]^ proves to be advantageous compared to Si (1.67 × 10^−2^ S m^−1^ and ranging from 10^−14^ to 10^−10^ cm^2^ s^−1^) for high‐power LIBs.^[^ ^6^, ^7^ ^]^ Si and Ge materials, however, face challenges such as the continuous formation of solid electrolyte interface (SEI) films, electrode fracture, and unstable electrical contact with the electrolyte,^[^ ^8^, ^9^ ^]^ attributed to significant volume expansion (≈300 to 400% and ≈200 to 250%, respectively) during battery cycling, ultimately leading to capacity degradation.^[^ ^10^, ^11^ ^]^

The recent shift in focus toward silicene and germanene, 2D counterparts of Si and Ge, as analogs of graphene in the group IV family,^[^ ^12^, ^13^ ^]^ reflects ongoing research efforts in exploring novel materials for advanced battery technologies. By introducing covalently bonded hydrogen in the z‐orientation of each atom in silicene and germanene, the bonding sites undergo hybridization changes from sp^2^ to sp^3^, resulting in the formation of hydrogen‐terminated silicene (SiH) and hydrogen‐terminated germanene (GeH).^[^ ^14^, ^15^ ^]^ It should be noted that SiH and GeH cannot be directly obtained from their bulk counterparts due to their diamond cubic crystal structure.^[^ ^16^ ^]^ The current synthetic methods primarily rely on “top‐down” chemical exfoliation from so‐called Zintl phases (CaSi_2_, CaGe_2_, and EuGe_2_).^[^ ^17^ ^]^ Subsequently, the removal of the calcium or europium interlayer using hydrochloric acid at low temperatures can lead to the acquisition of the targeted materials.^[^ ^18^, ^19^, ^20^ ^]^

Si and Ge exhibit complete miscibility in various ratios, prompting comprehensive studies on SiGe alloys in rechargeable batteries.^[^ ^21^, ^22^, ^23^ ^]^ However, limited attention has been given to their 2D derivatives, such as silicane‐germanane alloys (Si*_x_* Ge_1−_ *_x_* H). The preparation of these alloys is valuable to understanding the tunability of their electronic structure and electrochemical properties.^[^ ^24^, ^25^ ^]^ Theoretically, the 2D honeycomb structure of Si*_x_* Ge_1−_ *_x_* H is energetically stable due to the similar covalent radii of Si and Ge atoms, allowing for geometric adaptation to different atomic configurations.^[^ ^26^, ^27^ ^]^ By adjusting the *x* value, their electronic and electrochemical properties can be modulated, with expected band gaps between 1.09 and 2.29 eV for 0 ≤ *x* ≤1.^[^ ^28^ ^]^ While the potential of SiH or GeH as electrode materials in LIBs has been demonstrated,^[^ ^29^, ^30^, ^31^, ^32^ ^]^ the electrochemical mechanisms of Si*_x_* Ge_1−_ *_x_* H have not been extensively studied. Factors such as structure, particle morphology and cycling conditions known to affect lithiated species formation in Si and Ge,^[^ ^33^, ^34^ ^]^ are likely influential in Si*_x_* Ge_1−_ *_x_* H.

Amorphous materials in contrast to crystalline ones offer advantages.^[^ ^35^, ^36^ ^]^ These include reduced volume strain, resulting in better electrode integrity during the charge/discharge process,^[^ ^37^, ^38^ ^]^ easier structural restoration during electrochemical reactions due to the absence of grain boundaries and lower formation energy,^[^ ^39^, ^40^ ^]^ and faster Li^+^ diffusion pathways through the creation of active diffusion channels, leading to improved reaction kinetics.^[^ ^41^ ^]^ Prior studies have indicated that defect engineering through dehydrogenation creates defect sites on the surface of the amorphous materials, enhancing their conductivity.^[^ ^42^ ^]^ In this work, amorphous Si*_x_* Ge_1−_ *_x_* H, obtained via a chemical etching process from the Zintl phase Ca(Si*_x_* Ge_1−_ *_x_*)_2_ and subsequently washed with HF solution, introduces defect‐rich sites that reduce the crystallinity of Si*_x_* Ge_1−_ *_x_* H.^[^ ^43^ ^]^ In addition, the advantages of the short diffusion path of electrons/ions and the good mechanical flexibility arising from the loose‐layered structures of amorphous materials contribute to enhancing their cycling stability in batteries.^[^ ^44^ ^]^

Following this trend, the Si*_x_* Ge_1−_ *_x_* H materials with varying compositions (*x* = 0.25, 0.50, and 0.75) were synthesized through a topochemical deintercalation route. These materials, primarily characterized by a blend of amorphous and crystalline phases, exhibit substantial surface area, exceptional mechanical flexibility, swift electron mobility, and feature defect‐rich, loosely layered structures.^[^ ^45^ ^]^ The Si_0.50_Ge_0.50_H electrode, in particular, demonstrates the highest capacity and promising rate performance. Density functional theory (DFT) calculations were employed to clarify the electronic band structure, lithium‐ion diffusion pathways, and energy barriers within the defect‐rich Si_0.50_Ge_0.50_H. To understand the lithium storage mechanism, various experimental techniques were used such as ex‐situ transmission electron microscopy (TEM), high‐resolution TEM (HRTEM), and selected area electron diffraction (SAED). Additionally, ex‐situ Raman spectroscopy, X‐ray diffraction (XRD), and scanning electron microscopy (SEM) with energy‐dispersive X‐ray spectroscopy (SEM‐EDX) were employed to study phase conversions during battery cycling.

## 2 Results and Discussion

**Figure 1:**
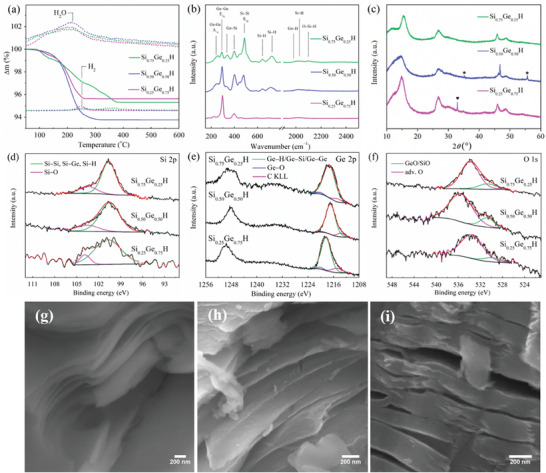
a) TG‐MS analysis (the dashed lines reflect the corresponding mass (m/z), whereas the full lines represent mass loss), b) Raman spectra, c) XRD patterns, and XPS spectra of d) Si 2*p*, e) Ge 3*d*, and f) O 1*s* core levels of Si_0.25_Ge_0.75_H, Si_0.50_Ge_0.50_H, and Si_0.75_Ge_0.25_H, side‐view SEM images of g) Si_0.25_Ge_0.75_H, h) Si_0.50_Ge_0.50_H, and i) Si_0.75_Ge_0.25_H.

Ca etching resulting from the topotactic deintercalation of Ca(Si*_x_* Ge_1−_ *_x_*)_2_ (*x* = 0.25, 0.5, and 0.75), led to the formation of Si*_x_* Ge_1−_ *_x_* H. A comprehensive series of material characterizations were conducted to gain insights into their structural, surface, and electrochemical properties. As depicted in **Figure** **1**a, the mass loss at 100 °C represents water. Since the hydroxyl groups (OH) are mainly bonded to silicon (Si–OH) rather than Ge, with higher Si content, the water loss is more rapid, which we believe, is the cause of the different slope of the line representing Si_0.75_Ge_0.25_H. At 180 °C, hydrogen loss becomes significant for higher Ge content, so the loss becomes more rapid for Si_0.50_Ge_0.50_H and Si_0.25_Ge_0.75_H. During the continuous thermal decomposition and combustion of the Si*_x_* Ge_1−_ *_x_* H samples, a significant amount of H_2_O and H_2_ was released. Si_0.25_Ge_0.75_H experiences a mass loss of ≈4.4% within the temperature range of 100–260 °C, which most likely can be attributed to the decomposition of H_2_O,^[^ ^46^ ^]^ together with the hydrogen, occurring at the edges and in the plane of the framework.^[^ ^47^ ^]^ The thermal decomposition of Si_0.50_Ge_0.50_H and Si_0.75_Ge_0.25_H demonstrate a mass loss of ≈6.2% and ≈4.7% within the temperature range of 100–320 °C and 100–385 °C, respectively, which could be ascribed to the loss of H_2_O and hydrogen, associated with the decomposition from the edges and in the plane of the framework as well.

Figure 1b depicts the Raman shifts of Si*_x_* Ge_1−_ *_x_* H. The characteristic peak at ≈250 cm^−1^ emerges in the spectra of all samples, which is identified as the Ge−Ge in‐plane A_1g_ vibrational mode.^[^ ^48^ ^]^ In the spectrum of Si_0.25_Ge_0.75_H, a strong and the most dominant peak corresponding to the E_2g_ vibrational mode of Ge−Ge is observed at ≈290 cm^−1^, while Si_0.50_Ge_0.50_H exhibits a relatively small peak at the same position. The peak intensity of the E_2g_ (Ge−Ge) vibrational mode weakens in Si_0.75_Ge_0.25_H in comparison to the other samples. Additionally, a novel peak is identified at ≈400 cm^−1^ in all samples, which corresponds to the vibrational mode of Ge−Si bonds.^[^ ^49^ ^]^ For Si_0.50_Ge_0.50_H and Si_0.75_Ge_0.25_H, a new E_2g_ (Si−Si) vibrational mode is observed at 480 cm^−1^, along with peaks at ≈640 and 715 cm^−1^, which are assigned to the Si−H vibrational modes. Furthermore, three weak peaks are observed in Si_0.50_Ge_0.50_H and Si_0.75_Ge_0.25_H at ≈1980−2200 cm^−1^, corresponding to the vibrational modes of Ge−H, Si−H, and O−Si−H. An interesting observation is that the vibrational mode of the Si−H peak becomes apparent only when *x* exceeds 0.5. As the value of *x* increases, both the Ge−Ge and Si−Si peaks exhibit broadening.

Figure 1c contains XRD scans of Si*_x_* Ge_1−_ *_x_* H, revealing broad diffraction peaks. Based on this observation, it can be inferred that the exfoliated materials consist predominantly of mixed amorphous and crystalline phases, considering the number of layers and interlayer distances.^[^ ^50^ ^]^ Specifically, for Si_0.25_Ge_0.75_H, the predominant peak occurs at 14.8° (≈5.97 Å), corresponding to the (002) plane, while for Si_0.50_Ge_0.50_H, it appears at 14.7° (≈6.01 Å), and for Si_0.75_Ge_0.25_H, it is observed at 15.5° (≈5.72 Å). The width of the peaks suggests that the materials are highly distorted and have a high degree of exfoliation.^[^ ^51^ ^]^ Additionally, there is a significant reflection occurring at 26.8° (≈3.32 Å), 26.9° (≈3.31 Å), and 26.8° (≈3.32 Å) for the three samples, corresponding to the (100) plane, respectively. Peaks at 46.4 and 48.8° correspond to the (110) and (112) planes. It is noteworthy that the large interlayer spacing, facilitating Li^+^ ion diffusion and improving accessibility to active sites, provides the biggest space for Li^+^ ion alloying.^[^ ^52^ ^]^

The chemical bonding of the Si*_x_* Ge_1−_ *_x_* H samples was confirmed through FTIR analysis. As depicted in Figure S1a (Supporting Information), Si_0.25_Ge_0.75_H displays two prominent peaks at ≈2005 and ≈485 cm^−1^, which can be identified as stretching and wagging Ge−H vibrational modes. The peaks at ≈910−590 cm^−1^ are associated with SiH_2_ edge termination, Si–O, Si–H bending and wagging vibrations. The peak at ≈1044 cm^−1^ is linked to the Si−O−Si vibrational mode. The Ge−H bond experiences a shift to higher wavenumbers in Si_0.50_Ge_0.50_H and Si_0.75_Ge_0.25_H. In what concerns the Si−H stretching vibration peak at 2100 cm^−1^, it is only observed in Si_0.50_Ge_0.50_H and Si_0.75_Ge_0.25_H. Furthermore, the Si−H stretching vibration peak becomes more prominent than the Ge−H wag in Si_0.75_Ge_0.25_H. Due to oxidation in the samples, a minor peak at ≈2240 cm^−1^ is observed in all Si*_x_* Ge_1−_ *_x_* H samples, which is attributed to the formation of O_3_Si–H bonding.^[^ ^53^ ^]^

To investigate the electronic states and conduct quantitative surface elemental analysis in the Si*_x_* Ge_1−_ *_x_* H samples, XPS measurements were carried out. Figure S1b (Supporting Information) shows the survey spectra of Si_0.25_Ge_0.75_H, Si_0.50_Ge_0.50_H, and Si_0.75_Ge_0.25_H, indicating the presence of Si, Ge, C, and O elements. The high‐resolution Si 2p spectra of Si_0.25_Ge_0.75_H exhibit peaks at ≈103 and 106 eV, corresponding to Si−Si/Si−Ge/Si−H and Si−O bonding states, respectively (Figure 1d). Similar peaks are observed in the Si 2p spectra of Si_0.50_Ge_0.50_H and Si_0.75_Ge_0.25_H, with binding energies of ≈101 and 104 eV. In the high‐resolution Ge 2p spectra of Si_0.50_Ge_0.50_H, peaks at ≈1217 eV suggest the presence of Ge−Ge/Ge−Si/Ge−H bonds (Figure 1e). Additionally, shoulder peaks at ≈1220 eV correspond to Ge−O bonds, likely attributed to surface oxidation. Similar peaks at 1218 and 1220 eV are observed in the Ge 2p spectra of Si_0.25_Ge_0.75_H and Si_0.75_Ge_0.25_H, as depicted in Figure 1e. The high‐resolution O 1s spectra of Si_0.50_Ge_0.50_H in the 529–540 eV range reveal the states of surface oxygen (Figure 1f). Peaks at 531 and 536 eV indicate the presence of GeO/SiO and adventitious O, respectively. Similarly, peaks at 530 and 534 eV, as well as peaks at 531 and 534 eV, are observed in the spectra of Si_0.25_Ge_0.75_H and Si_0.75_Ge_0.25_H, correspondingly. It can be concluded that the high‐resolution Si 2p, Ge 2p, and O 1s spectra of all samples exhibit a similar pattern, with slight variations depending on the Si ratio.

SEM imaging depicts a layered morphology for the exfoliated Si*_x_* Ge_1−_ *_x_* H samples, exhibiting variations in lateral dimensions and layer numbers. Upon closer examination of the surface morphology, the distinctive 2D defect‐rich loose‐layered structure inherent in all three samples becomes evident. The top (Figure S1c–e, Supporting Information) and side view (Figure 1g–i) display the loose‐layered morphology of the materials. The composition of each sample was verified through EDX analysis (see Figure S2, Supporting Information). The elemental mapping and corresponding EDX spectra images reveal a homogeneous distribution of Si and Ge elements in the samples. The presence of oxygen is attributed to the slight oxidation of the prepared sample. The residual traces of Cl and F elements are associated with the utilization of HCl and HF during the preparation of Si*_x_* Ge_1−_ *_x_* H. According to the EDX analysis, the molar ratios of Si to Ge are ≈1:3.74, 1:1.29, and 3.05:1, aligning well with the expected ratio.

BET analysis reveals changes in specific surface area and total pore volume. Si_0.25_Ge_0.75_H, Si_0.50_Ge_0.50_H, and Si_0.75_Ge_0.25_H exhibit specific surface areas of 21.26, 16.14, and 81.99 m^2^ g^−1^, respectively, as shown in Table S1 (Supporting Information). In principle, a higher surface area provides more reaction sites for ion migration, ensuring sufficient electrode‐electrolyte contact.^[^ ^54^ ^]^ As it can be seen in Figure S3a (Supporting Information), the Si_0.75_Ge_0.25_H material shows a porous structure evident in adsorption–desorption isotherms. This is corroborated by the pore size distribution curve in Figure S3b (Supporting Information), revealing predominantly non‐uniformly distributed pores within a range smaller than 4 nm. On the other hand, the presence of mesoporous structures in the three samples can shorten Li^+^ ion diffusion paths and reduce volume changes during the charge/discharge process, improving electrochemical kinetics.^[^ ^55^ ^]^

**Figure 2:**
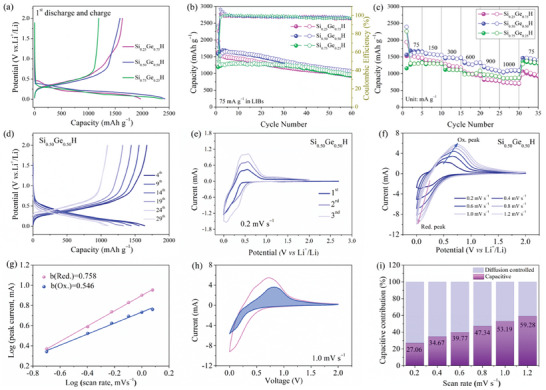
Cycling performance of Si_0.25_Ge_0.75_H, Si_0.50_Ge_0.50_H and Si_0.75_Ge_0.25_H in lithium coin cells galvanostatically cycled at a current density of 75 mA g^−1^ in terms of a) voltage profiles at the 1^st^ cycle and b) specific capacity vs. cycling number. Rate capacities at current densities of 75, 150, 300, 600, 900, and 1000 mA g^−1^ in terms of c) specific capacity vs. cycling number and d) voltage profiles of Si_0.50_Ge_0.50_H at selected cycles. e) Initial 3‐cycle CV curves at 0.2 mV s^−1^; f) CV at 0.2–1.2 mV s^−1^; g) relationship between log (peak current) and log (scan rate); h) capacitive and diffusion‐controlled contributions at 1.0 mV s^−1^; i) normalized capacitive contribution and the diffusion‐controlled contribution fractions at varying scan rates of Si_0.50_Ge_0.50_H.

The electrochemical properties of the Si*_x_* Ge_1−_ *_x_* H electrodes in lithium half‐cells were investigated by coupling galvanostatic measurements and cyclic voltammetry (CV), as shown in **Figure** **2**. First, the electrodes were subjected to galvanostatic cycling in CR2025‐type coin half‐cells, with a current density of 75 mA g^−1^ and a voltage window of 0.01−2.0 V vs. Li^+^/Li. Figure 2a illustrates the 1^st^ representative galvanostatic discharge/charge voltage profiles. Notably, the Si_0.50_Ge_0.50_H electrode exhibits a discharge and charge capacity of 2419 and 1586 mAh g^–1^ (34%), compared to 1968 and 1632 mAh g^–1^ (17%) for Si_0.25_Ge_0.75_H as well as 2371 and 1192 mAh g^–1^(50%) for Si_0.75_Ge_0.25_H. Such an irreversible capacity loss is primarily attributed to the formation of the SEI layer on the electrode surface, the lithiation reaction induced by bulk impurities like Si or Ge, and the presence of functional groups between the layers.^[^ ^56^ ^]^ This observation aligns with the fact that Ge undergoes lower volume expansion during lithiation and delithiation than Si, which helps to mitigate the initial capacity loss.^[^ ^57^ ^]^ Si_0.25_Ge_0.75_H and Si_0.50_Ge_0.50_H both exhibit pronounced gradual voltage slopes ranging from 0.46 to 0.19 V and from 0.39 to 0.22 V during discharge, as well as extensive plateaus persisting below 0.19 V and below 0.22 V. Si_0.75_Ge_0.25_H displays a significant gradual slope between 0.39 and 0.12 V, accompanied by a lengthy plateau at approximately 0.12 V. In these three electrodes, the sloping region reflects the amorphization of c‐Si*_x_* Ge_1−_ *_x_* to form *a*‐Li*_y_* (Si*_x_* Ge_1‐_ *_x_*), while the plateau region at low potential corresponds to the crystallization of *c*‐Li_15_(Si*_x_* Ge_1‐_ *_x_*)_4_.^[^ ^13^ ^]^ Among them, Si_0.50_Ge_0.50_H exhibits a larger proportion of plateau region at low potential, indicating that the removal of H atoms allows more Li⁺ ions to alloy into this material. Consequently, the Si_0.50_Ge_0.50_H electrode is anticipated to achieve a high capacity and promising rate performance. It is worth noting that a more accurate description for the composition of the discussed material would be amorphous Si*_x_* Ge_1−_ *_x_* due to the structural transformation that took place during the first Si*_x_* Ge_1−_ *_x_*‐Li alloying/dealloying reaction.

Figure 2b illustrates the corresponding capacity trend, showing variations in capacity over 60 cycles. During the first 22 cycles, the specific capacity of Si_0.25_Ge_0.75_H is higher than Si_0.75_Ge_0.25_H, but the capacities decay rapidly as the cycle increases. The Si_0.75_Ge_0.25_H electrode demonstrates a low initial Coulombic efficiency (ICE) of 50%, compared to 83% for Si_0.25_Ge_0.75_H and 66% for Si_0.50_Ge_0.50_H. The specific capacity gradually increases as the cycle progresses, reaching 1259 mAh g^−1^ in the second cycle and 1247 mAh g^−1^ after 30 cycles, indicating the gradual activation of Si_0.75_Ge_0.25_H. However, the specific capacity of Si_0.50_Ge_0.50_H remains slightly higher than Si_0.75_Ge_0.25_H throughout the cycling process. The discharge capacity of Si_0.50_Ge_0.50_H is maintained at 1059 mAh g^−1^ after 60 cycles, surpassing that of Si_0.25_Ge_0.75_H and Si_0.75_Ge_0.25_H, which are at 964 mAh g^−1^. This difference can be ascribed to the elevated Si content in Si_0.50_Ge_0.50_H and Si_0.75_Ge_0.25_H, which improves their specific capacity and cycling stability.^[^ ^58^, ^59^ ^]^ On the other hand, the capacity fading of the three electrodes can be attributed to the repeated alloying/dealloying of Li^+^ ions, causing excessive volume expansion and irreversible damage to the materials, thereby compromising their electrochemical properties.^[^ ^24^ ^]^ While Si_0.25_Ge_0.75_H initially exhibits a higher specific capacity and best Coulombic efficiency at the first cycle, as the cycling progresses, it experiences poor cycle stability and a significant decline in specific capacity, ultimately falling behind those of Si_0.50_Ge_0.50_H and Si_0.75_Ge_0.25_H.

The rate capability of the Si*_x_* Ge_1−_ *_x_* H electrodes was assessed, with each electrode undergoing 5 cycles at various current densities. Starting at 75 mA g^−1^, the current density was incrementally increased to 1000 mA g^−1^ before being returned to 75 mA g^−1^ (Figure 2c). For Si_0.25_Ge_0.75_H, regardless of the rate, continuous capacity decay occurs, likely due to some bulk Si or Ge materials (as seen in XRD, Figure 1c), triggering new alloying reactions and resulting in capacity decay. The discharge capacities of Si_0.75_Ge_0.25_H are 1298, 1290, 1113, 1006, 925, and 876 mAh g^−1^ at current densities of 75, 150, 300, 600, 900, and 1000 mA g^−1^, respectively. The discharge capacities of Si_0.50_Ge_0.50_H are 1641, 1561, 1443, 1329, 1107, and 1106 mAh g^−1^, which are higher than those of Si_0.25_Ge_0.75_H (1488, 1364, 1213, 979, 808, and 721 mAh g^−1^) at the same current densities. The 31^st^ cycle discharge capacity of Si_0.50_Ge_0.50_H at 75 mA g^−1^ is 1383 mAh g^−1^, while it increases to 1405 mAh g^−1^ in the 35^th^ cycle. This promising electrochemical performance can be attributed to the unique 2D defect‐rich loose‐layered structure, which effectively shortens Li^+^ ion diffusion pathways, accelerates interfacial charge transfer, and enhances the electrolyte/electrode interface stability.^[^ ^60^ ^]^ The corresponding discharge and charge curves of Si_0.50_Ge_0.50_H are presented in Figure 2d, showing very slight polarization as the current density is incrementally increased to 300 mA g⁻¹ and a moderate degree of polarization up to 1000 mA g⁻¹.

The long‐term cycling performance of Si_0.50_Ge_0.50_H was measured at high current densities of 1000 and 2000 mA g^–1^ over 1000 cycles, as depicted in Figure S4 (Supporting Information). It maintains a discharge capacity exceeding 1000 mAh g^–1^ for the initial 40 cycles at 1000 mA g^–1^, decreasing to below 300 mAh g^–1^ after 120 cycles. At 2000 mA g^–1^, it exhibits a specific capacity of 240 mAh g^–1^ after 120 cycles. Following 1000 cycles, the residual capacities of Si_0.50_Ge_0.50_H at current densities of 1000 and 2000 mA g^–1^ are 128 and 75 mAh g^–1^, respectively. The suboptimal performance is assigned to poor conductivity and structural damage induced by substantial volume changes in the active nanosheets. Consequently, we propose enhancing the cycling stability of the Si_0.50_Ge_0.50_H electrode through modification via shear force exfoliation and carbon coating.

The volume changes in the electrode materials, particularly Si can lead to the formation of a SEI layer, affecting the overall consumption of electrolytes during cycling. The specific proportion of Si and Ge in the Si*_x_* Ge_1−_ *_x_* H alloy can influence the magnitude of these volume changes. Research work indicated that a higher silicon content in the alloy tends to result in more significant volume changes, potentially causing increased electrolyte consumption.^[^ ^61^ ^]^ To investigate volumetric changes in the electrode materials under a current density of 600  mA g^–1^ after 60 cycles, the cells were disassembled within an argon‐filled glovebox, and the electrodes were washed with DMC solvent. Figure S5 (Supporting Information) illustrates cross‐sectional SEM images of the Si_0.25_Ge_0.75_H, Si_0.50_Ge_0.50_H, and Si_0.75_Ge_0.25_H electrodes before and after cycling. It is evident from Figure S5d,f (Supporting Information) that the Si_0.25_Ge_0.75_H and Si_0.75_Ge_0.25_H electrodes, after cycling, display thicknesses of ≈320 µm and 135 µm, corresponding to volumetric expansions of ≈4 and 2.7 times, respectively. Figure S5e (Supporting Information) indicates volume expansion of Si_0.50_Ge_0.50_H by a factor of ≈1.8. The respective energy‐dispersive EDX spectra and corresponding elemental mapping before and after cycling are depicted in Figure S6 (Supporting Information). The atomic ratio values remain similar after cycling, indicating a small segregation of Si or Ge elements. The elemental mapping of Si and Ge is evenly distributed on the electrode, both before and after cycling. Accordingly, it can be reasonably concluded that the Si_0.50_Ge_0.50_H electrode maintains structural integrity under this volumetric expansion rate, likely thanks to the favorable Si/Ge ratio and synergistic effects between Si and Ge.

XPS analysis was applied to detect the primary element components, specifically Li and F, on the surfaces of Si_0.25_Ge_0.75_H, Si_0.50_Ge_0.50_H and Si_0.75_Ge_0.25_H electrodes after 60 cycles. The absence of discernible peaks in the Li 1s and F 1s spectra of the electrode before cycling is evident, as depicted in Figure S7a,c (Supporting Information). The Li 1s XPS spectra of the three Si_0.25_Ge_0.75_H, Si_0.50_Ge_0.50_H, and Si_0.75_Ge_0.25_H electrodes, following 60 cycles, as illustrated in Figure S7b (Supporting Information), manifests a peak at 55.6 eV, attributed to Li_2_CO_3_,^[^ ^62^, ^63^ ^]^ accompanied by three peaks at 56.6, 54.6 and 53.6 eV, which may be ascribed to LiF/LiPF_6_/LiPO_x_F_y_, LiOH and Li_2_O.^[^ ^64^, ^65^ ^]^ The emergence of peaks at 685.4 and 687.7 eV in the F 1s XPS spectrum after 60 cycles (Figure S7d, Supporting Information) indicates the presence of LiF and LiPF_6_/LiPO_x_F_y_.^[^ ^66^, ^67^ ^]^ It needs to be noted that the data reported herein present a rather qualitative character. For the Li 1s region, a wide range of binding energies can be found in the literature where e.g., Li_2_O has binding energies reported from 53.5 eV to nearly 56 eV.^[^ ^68^ ^]^ Similarly, for other Li compounds such as Li_2_CO_3_, LiF, LiPF_6_, etc. the reported binding energies fall within a wide range, which makes any reliable identification and quantification extremely difficult. The peak intensity of Li 1s and F1s in the Si_0.50_Ge_0.50_H electrode was lower than that in the Si_0.25_Ge_0.75_H and Si_0.75_Ge_0.25_H electrodes, suggesting a significant reduction in side reactions with the electrolyte. Consequently, based on the XPS results, it can be inferred that after cycling the SEI film is formed through the electrolyte decomposition, primarily involving the generation of LiF, LiPF_6_/LiPO_x_F_y_, Li_2_O, LiOH and Li_2_CO_3_. It can be inferred that an optimal silicon content (*x* = 0.5) enhances the stability and ionic conductivity of the SEI layer, which contributes to the suppression of lithium dendrites and facilitates lithium‐ion transport, ultimately leading to stable cycling performance.^[^ ^69^, ^70^ ^]^

Figure 2e illustrates the voltammetry profiles of the Si_0.50_Ge_0.50_H electrode in a three‐electrode lithium cell, recorded over three cycles at a scan rate of 0.2 mV s^−1^, within a potential window of 0.01−2.0 V vs. Li^+^/Li. During the first cycles, one broad reduction peak becomes apparent from 0.39 to 0.01 V, which can be attributed to the formation of SEI films, and the alloying reaction between Si_0.50_Ge_0.50_H and Li^+^ ions, resulting in the formation of *c*‐Li_15_(Si*_x_* Ge_1‐_ *_x_*)_4_.^[^ ^71^, ^72^ ^]^ The dealloying process takes place, marked by two wide oxidation peaks at 0.41 and 0.55 V, ascribed to the delithiation of *c*‐Li_15_(Si*_x_* Ge_1‐_ *_x_*)_4_ and amorphous Si_0.50_Ge_0.50_ formation.^[^ ^73^ ^]^ In the second cycle, the reduction peak is substituted by a broad peak that is located between 0.54 and 0.01 V. Additionally, there is an increase in the intensity of the oxidation peaks at 0.41 and 0.55 V, denoting the activation of a bigger portion of the Si_0.50_Ge_0.50_H.^[^ ^74^ ^]^ The weak reduction peak at 0.06 V gains intensity in the third cycle. The reduction peak at 0.54 V gradually shifts to 0.63 V, and the oxidation peaks at 0.41 and 0.55 V move to 0.44 and 0.57 V, constantly increasing in intensity. Throughout the cycling process, the intensity of the redox peaks gradually increased, indicating a progressive activation of the Faradaic electrochemical reaction.^[^ ^24^ ^]^ This may be attributed to the favorable electrical conductivity of Ge and the distinctive 2D structure of defect‐rich loose‐layered Si_0.50_Ge_0.50_H, which enhances the contact between the electrolyte and electrode.^[^ ^24^ ^]^ Figure S8 (Supporting Information) displays the voltammetry profiles of the Si_0.25_Ge_0.75_H and Si_0.75_Ge_0.25_H electrodes.

The intricate reaction kinetics of the Si_0.25_Ge_0.75_H, Si_0.50_Ge_0.50_H, and Si_0.75_Ge_0.25_H electrodes after 60 cycles were investigated through electrochemical impedance spectroscopy (EIS). Employing Nova 2.1, the impedance spectra were meticulously analyzed. The Nyquist plots (Figure S9a, Supporting Information) predominantly exhibit a sloping line and a semicircle in the low and middle frequencies, along with two semicircles in the high‐frequency region, which can be fitted using a Randles equivalent circuit, as illustrated in Figure S9b (Supporting Information). Within this circuit, R_s_ represents electrolyte resistance, R_SEI_ signifies the resistance of the solid electrolyte interphase, R_ct_ denotes the charge transfer resistance, and W_s_ pertains to the Warburg impedance associated with the diffusion of Li^+^ ions into the bulk materials. The third semicircle (R_4_) might represent another electrochemical process, which could be associated with double‐layer capacitance (C_dl_), Warburg impedance (W_s_), or other phenomena. The corresponding fitted parameters are listed in Table S2 (Supporting Information). The R_ct_ values for Si_0.25_Ge_0.75_H, Si_0.50_Ge_0.50_H, and Si_0.75_Ge_0.25_H are measured at 15.07, 12.05, and 7.891 Ω respectively, indicating fast reaction kinetics at the three electrode‐electrolyte interfaces. This finding suggests that an optimized Si and Ge ratio not only enhances conductivity but also augments charge transfer efficiency, facilitating Li^+^ ion diffusion, and thereby corroborating its promising electrochemical performance.^[^ ^75^ ^]^

To investigate the charge storage mechanism and understand the favorable rate performance of the Si_0.50_Ge_0.50_H electrode, CV profiles were evaluated at different scan speeds ranging from 0.2 to 1.2 mV s^−1^. Despite slight variations, the different CV curves exhibit a similar shape characterized by broadening reduction and oxidation peaks, as depicted in Figure 2f. During a linear scan, the overall stored charge (and thus the current density or capacity) originates from two types of processes: a diffusion‐controlled process and an ideal capacitive behavior, both of which follow the power‐law described below:

(1) $$i=a^{v}b$$

In the equation, *i* and *v* represent the measured current and scan rate, respectively, while *a* and *b* are variable constants. A diffusion‐controlled process is characterized by *b* = 0.5, while an ideal capacitive behavior is represented by *b* = 1.0.

The relationship between log (peak current) and log (scan rate) observed in various CV profiles is depicted in Figure 2g. The reduction peak exhibits a slope of 0.758, indicating a comparatively higher contribution of capacitance‐controlled behavior. Conversely, the oxidation peak demonstrates a slope of 0.546, suggesting a predominantly diffusion‐controlled process. The observed asymmetric redox reactions can be attributed to the slower dealloying of Li^+^ compared to its alloying. The b values associated with the peaks during discharge are greater than their counterparts in the charging process. This indicates a more pronounced influence of capacitive control, specifically pseudocapacitive behavior.

The coexistence of the two processes mentioned above is characteristic of a real electrode reaction, and their contributions can be determined as outlined below:

(2) $$i(V)=k_{1}v+k_{2}v^{0.5}$$

In this equation, *k*_1_ *v* and *k*_2_ *v* ^0.5^ represent capacitance‐controlled and diffusion‐controlled contributions, respectively. The parameters *k*_1_ and *k*_2_ are obtained by transforming the equation provided below:

(3) $$\frac{i(V)}{{v^{0}}^{.5}}=k_{1}{v^{0}}^{.5}+k_{2}$$

*k*_1_ and *k*_2_ represent the slope and *y*‐intercept of the curve *i*(*V*)/*v* ^0.5^≈*v* ^0.5^, respectively. By adjusting the *v* value to 1.0 mV s^−1^, the capacitance‐controlled contribution is found to be 53.19%, as depicted in Figure 2h. This finding signifies the predominance of a Li^+^ storage mechanism involving surface pseudocapacitance, facilitating rapid and stable lithium storage capabilities, particularly at high current densities. Figure 2i illustrates the normalized contribution ratio of capacitive and diffusion‐controlled capacities. The corresponding capacitance‐controlled contribution increases from 27.06, 34.67, 39.77, 47.34, 53.19 to 59.28% with an enhancement in the scan rate from 0.2 to 1.2 mV s^−1^, highlighting the outstanding rate capability of Si_0.50_Ge_0.50_H. These results corroborate that the capacitance‐controlled process exerts a pivotal influence on the lithium storage characteristics in the Si_0.50_Ge_0.50_H electrode, likely owing to the presence of a 2D defect‐rich loose‐layered structure. This structural feature offers numerous active surface sites, shorter Li‐ion diffusion pathways and faster electronic transmission routes.^[^ ^76^ ^]^ Consequently, the aforementioned analyses furnish compelling evidence supporting the optimistic rate capability of Si_0.50_Ge_0.50_H.

Table S3 (Supporting Information) displays the recently reported electrochemical properties of SiGe‐based anode materials for LIBs, allowing an easy visual comparison. Notably, the cycling stability of Si_0.50_Ge_0.50_H in our study surpasses that reported in recent literature for materials such as Si_0.50_Ge_0.50_ (1142 mAh g^–1^ at a current density of 50 mAh g^–1^ after 10 cycles)^[^ ^24^ ^]^ and Ge_0.1_Si_0.9_ (1020 mAh g^–1^ at C/2 after 100 cycles).^[^ ^25^ ^]^ Additionally, the initial coulomb efficiency of Si_0.50_Ge_0.50_H (66%) in our work exceeds that in recently reported literature for Si_0.50_Ge_0.50_ (48.61%).^[^ ^24^ ^]^

The disparity observed in the BET surface area and EIS results, favoring Si_0.75_Ge_0.25_H, while the promising cycle performance and volumetric expansion benefiting Si_0.50_Ge_0.50_H, can be attributed to various factors. The 0.5 ratio may benefit from a more favorable pore structure, facilitating electrolyte penetration and ion transport during cycling. Additionally, considering not all geometric surfaces provided by BET are electrochemically active, the 0.5 ratio may possess a higher proportion of accessible surfaces, contributing to enhanced capacity. The 0.5 ratio may also exhibit improved wetting properties, promoting uniform electrolyte distribution and better rate performance. To delve into the mechanisms behind this enhanced electrochemical performance of Si*_x_* Ge_1−_ *_x_* H, and considering the good performance of Si_0.50_Ge_0.50_H, together with the advantage of the presence of some amount of Ge in the electrode in terms of cost, we will primarily focus on that material, setting aside Si_0.25_Ge_0.75_H or Si_0.75_Ge_0.25_H.

**Figure 3:**
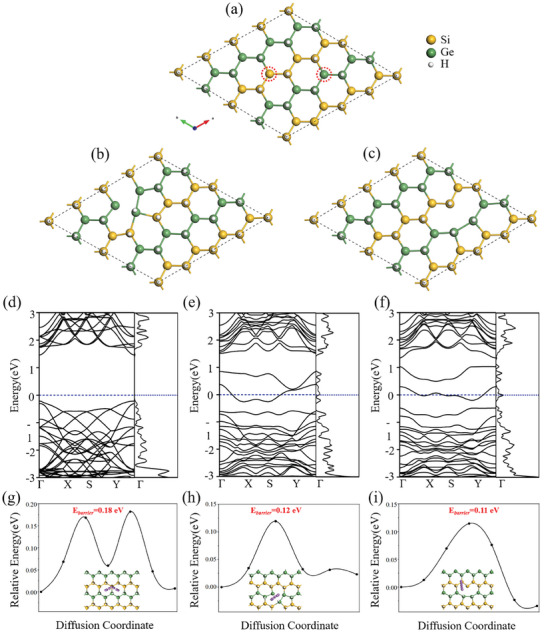
Atomic configuration of a) pristine Si_0.50_Ge_0.50_H, b) Si‐defects, c) Ge‐defects. DFT‐calculated electronic band structure of d) pristine Si_0.50_Ge_0.50_H, e) Si‐defects, f) Ge‐defects. Diffusion pathways and energy barriers of Li^+^ ions on g) pristine Si_0.50_Ge_0.50_H, h) Si‐defects, i) Ge‐defects.

Density Functional Theory (DFT) calculations have been employed to assess the atomic‐level stability as well as elucidate the electronic band structure, Li^+^ ion diffusion pathways and energy barriers of defect‐rich Si_0.50_Ge_0.50_H. The structure of pristine Si_0.50_Ge_0.50_H used in the present computational study holds a supercell of 16 Si, 16 Ge, and 32 H atoms, as illustrated in **Figure** **3**a. The calculated lattice parameter of pristine Si_0.50_Ge_0.50_H is 3.96 Å. As plotted in Figure 3a, the Si_0.50_Ge_0.50_H with crystal defects were modeled by removing one Si or Ge atom from the pristine Si_0.50_Ge_0.50_H supercells, and the optimized structures of defective Si_0.50_Ge_0.50_H are demonstrated in Figure 3b,c for Si‐defects crystal and Ge‐defects crystal, respectively.

The electronic band structures of pristine and defective Si_0.50_Ge_0.50_H are illustrated in Figure 3d–f. It indicates that the pristine Si_0.50_Ge_0.50_H shows semiconductor property with a direct bandgap (E_g_) of 1.68 eV at a high symmetric Γ‐point. However, for defective Si_0.50_Ge_0.50_H, either Si‐defective or Ge‐defective, the Fermi level shifts to the conduction band, as shown in Figure 3e,f. This confirms that the electronic conductivity of Si_0.50_Ge_0.50_H can be greatly enhanced by the presence of Si or Ge defects.

The diffusion pathways and the energy barriers of Li on pristine Si_0.50_Ge_0.50_H and Si or Ge‐defective Si_0.50_Ge_0.50_H are analyzed through the climbing‐image nudged elastic band (CINEB) method, as illustrated in Figure 3g–i. It can be seen that the diffusion path of Li^+^ ions on pristine Si_0.50_Ge_0.50_H is between two adjacent stable Li‐adsorption sites by overcoming a maximum energy barrier of 180 meV in a curved path. As for Si‐defects and Ge‐defects Si_0.50_Ge_0.50_H, the maximum energy barriers are 120 meV and 110 meV, respectively, which are lower than that of pristine Si_0.50_Ge_0.50_H. This suggests that lattice defects promote the rapid diffusion of Li^+^ ions in Si_0.50_Ge_0.50_H. These results are consistent with our galvanostatic cycling data, providing further confirmation that the defect‐rich Si_0.50_Ge_0.50_H exhibits promising cycling performance.

**Figure 4:**
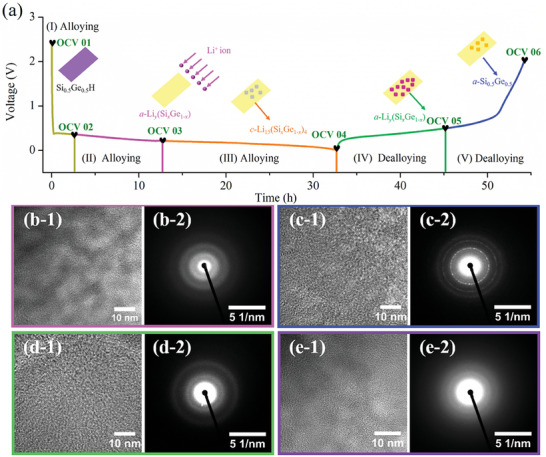
A schematic illustration of the reaction mechanism of the Si_0.50_Ge_0.50_H electrode at different stages a). Ex situ HRTEM, and corresponding SAED patterns of the Si_0.50_Ge_0.50_H electrode after 0 cycles b‐1,b‐2) discharged to 0.01 V c‐1,c‐2) charged to 2.0 V d‐1,d‐2) and after 25 cycles e‐1,e‐2).

Utilizing the revealed reaction sequence evident in the cyclic voltammetry (CV) curves presented in Figure 2e, we put forth a comprehensive 5‐stage scheme, illustrated in **Figure** **4**a. This schematic serves to elucidate the structural evolution and underlying reaction mechanisms associated with the Si_0.50_Ge_0.50_H electrode. To achieve a detailed understanding, the discharge/charge curves from the initial cycle were meticulously examined, leading to the identification of five distinct states (OCV 01 – OCV 06) for subsequent in‐depth investigation. The proposed five‐stage reaction mechanism for the operation of the Si_0.50_Ge_0.50_H electrode at various lithiation and delithiation stages is delineated as follows: Stage 1 (OCV–0.37 V); Stage 2 (0.37–0.21 V); Stage 3 (0.21–0.01 V); Stage 4 (0.01–0.5 V); Stage 5 (0.5–2.0 V). The figure incorporates color‐marked illustrations depicting the lithiation and delithiation processes corresponding to each phase composition. The resemblance between the cyclic voltammograms of SiGe alloys found in the literature implies that Si_0.50_Ge_0.50_H follows a similar electrochemical cycle.^[^ ^77^, ^78^, ^79^ ^]^ It experiences alloying reactions as follows:

(4) $$\begin{array}{ccc} & & \mathrm{Lithiation}:\;c-Si_{0.5}Ge_{0.5}H+\mathrm{Li}\to c-Si_{0.5}Ge_{0.5}+a\\ & & \;-Li_{y}\left(Si_{x}Ge_{1-x}\right)+-H\to c-Li_{15}{\left(Si_{x}Ge_{1-x}\right)}_{4} \end{array}$$

(5) $$\begin{array}{ccc} & & \mathrm{Delithiation}:\;c-Li_{15}{\left(Si_{x}Ge_{1-x}\right)}_{4}\to a-Li_{y}\left(Si_{x}Ge_{1-x}\right)\\ & & \;\to a-Si_{0.5}Ge_{0.5}+\mathrm{Li} \end{array}$$

To further support the aforementioned hypothesis from a structural evolution perspective, ex‐situ TEM, HRTEM, and SAED analyses were performed at various states. These analyses aim to investigate the structural changes that took place in the Si_0.50_Ge_0.50_H electrodes before and after cycling. The Si_0.50_Ge_0.50_H electrodes underwent a series of sequential processes, including 0 cycles, discharged to 0.01 V, charged to 2.0 V, and after 25 cycles. Before cycling, the TEM image (Figure S10a, Supporting Information) illustrates the 2D loose‐layered morphology of the visible flakes. HRTEM investigation (Figure 4b‐1) shows that Si_0.50_Ge_0.50_H is composed of a mixture of amorphous and crystalline phases, and the corresponding diffraction rings can be seen in Figure 4b‐2. Subsequently, following discharge to 0.01 V, TEM imaging indicates that some layers experienced exfoliation, resulting in smoother edges (Figure S10b). This smoothing effect is likely due to the formation of a passivation SEI layer on the *c*‐Li_15_(Si*_x_* Ge_1‐_ *_x_*)_4_ surface. Additionally, the SAED image displays a combination of amorphous and crystalline phases (Figure 4c‐2). After being charged to 2.0 V, TEM analysis reveals that the layered morphology of *a*‐Si_0.50_Ge_0.50_ remains distinguishable (Figure S10c). However, the edges of *a*‐Si_0.50_Ge_0.50_ become blurred. The HRTEM and SAED images depicted in Figure 4d‐1,d‐2, respectively, confirm the amorphous state of the *a*‐Si_0.50_Ge_0.50_. After 25 cycles, as shown in Figure S10d, the original morphology of *a*‐Si_0.50_Ge_0.50_ has partially disappeared, giving rise to a continuous porous network structure. Figure 4e‐1 depicts the corresponding HRTEM image, which reveals the absence of observable lattice fringes, indicating the amorphous nature of *a*‐Si_0.50_Ge_0.50_. The SAED image in Figure 4e‐2, lacking diffraction rings, confirms that Si_0.50_Ge_0.50_H has transformed into an amorphous state during cycling due to volume changes.

**Figure 5:**
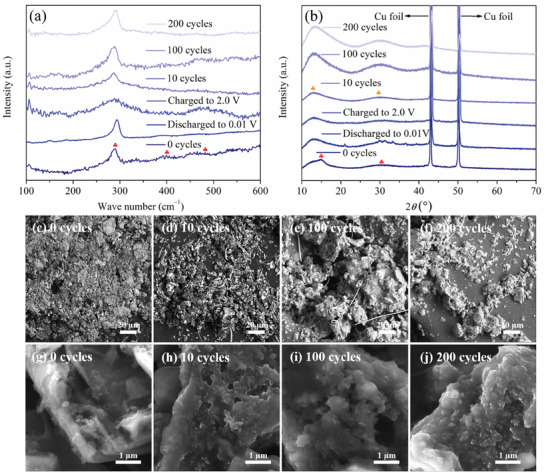
a) Ex‐situ Raman spectra and b) ex‐situ XRD patterns of Si_0.50_Ge_0.50_H during various lithiated and delithiated states. SEM images of Si_0.50_Ge_0.50_H at different magnifications after: 0 cycles c,g) 10 cycles d,h) 100 cycles e,i) and 200 cycles f,j).

Ex‐situ Raman spectroscopy, XRD, SEM and SEM‐EDX measurements were carried out to elucidate the lithiation/delithiation process after 0 cycles, 1 cycle (discharged to 0.01 V), 1 cycle (charged to 2.0 V), 10, 100, and 200 cycles. Phase changes and morphological evolution in Si_0.50_Ge_0.50_H during cycling are depicted in **Figure** **5**. The Raman spectrum of (Figure 5a) initially exhibits a broad peak centered at 290 cm^−1^ and two flat peaks at 400 and 480 cm^−1^, which are assigned to the Ge–Ge, Ge–Si, and Si−Si bonds. At the end of discharge at 0.01 V, the peak at 290 cm^−1^ slightly shifts to 294 cm^−1^, while at the end of charge at 2.0 V, it shifts to 288 cm^−1^. In addition, the peaks observed at 400 cm^−1^ disappear after the first discharge. Ge–Ge, Ge–Si, and Si−Si bands emerge and the peaks appear broader after the first charge, possibly indicating a reduction in particle size,^[^ ^80^ ^]^ thereby confirming the reformation of Si–Ge bonds. The shift observed in the Ge–Ge, Ge–Si, and Si−Si peaks during the initial discharge and charge processes could be associated with the phase transition of Si_0.50_Ge_0.50_H from mixed crystalline and amorphous to fully amorphous phases.^[^ ^29^, ^81^ ^]^ Importantly, the predominant Ge–Ge bonds remain noticeable throughout the entire duration, while Ge–Si and Si−Si bonds vanish after 10, 100, and 200 cycles. This disappearance could potentially be attributed to the low sensitivities of these modes and the reduced depth in the optical penetration following 10 cycles and subsequent cycles.

Figure 5b presents the related ex‐situ XRD patterns of Si_0.50_Ge_0.50_H upon electrochemical (de)lithiation. The two peaks observed at 42.7° and 49.8° correspond to the peaks of the Cu foil. The electrode, before cycling, maintains a consistent peak at 14.7°, while the peak at 26.9° shifts to 29.7°. The intensities of these humps progressively rise after discharge to 0.01 V, with the primary peak shifting into a broader peak located at 12.9°, while another peak remains steady at 29.7°. It is worth noting that these broad peaks are more likely associated with *c*‐Li_15_(Si*_x_* Ge_1‐_ *_x_*)_4_ phases that exhibit varying degrees of lithiation. It also shows some small new peaks (21.4, 31.4, 33.3, and 36.6°), which may be ascribed to the impurity of Li*_x_* Si or Li*_x_* Ge alloy intermediates. The XRD analysis shows that the two primary broad peaks are present in the electrode charged to 2.0 V, and they remain consistent in the subsequent cycles for up to 10, 100, and 200 cycles, without the appearance of the four small peaks. Si_0.50_Ge_0.50_H did not undergo significant structural changes from the first cycle up to 200 cycles, indicating a preferential lithiation of Li^+^ ions into some specific crystallographic SiGe planes. This observation suggests that Si_0.50_Ge_0.50_H as a silicane‐germanane alloy material follows a similar pattern, potentially containing delithiated *a*‐Si_0.50_Ge_0.50_ phase.^[^ ^30^ ^]^

The Si_0.50_Ge_0.50_H electrodes underwent a series of 0, 10, 100, and 200 half‐cell cycles, followed by ex‐situ SEM and SEM‐EDX analysis to evaluate the impact of lithiation/delithiation. Figure 5c–j depicts the observed morphological changes after various cycling processes. The original electrode material exhibits an inhomogeneous size distribution, characterized by multiple lateral sizes, and composed of stacked layers (see Figure 5g). The initial layered morphology of Si_0.50_Ge_0.50_H remains somewhat discernible due to the SEI layer formation and volume variations induced by lithiation after 10 cycles (Figure 5h). Nevertheless, after 100 cycles, the morphology of Si_0.50_Ge_0.50_H becomes less distinct (as depicted in Figure 5i), and a heightened level of deformation is noticeable. Few outlines of few‐layer structures are distinguishable at this stage, the formation of the surface SEI film becomes prominent, and some restructuring of Si_0.50_Ge_0.50_H takes place, leading to partial fusion with the surrounding SEI film. The components of the SEI films on the surfaces of the Si_0.50_Ge_0.50_H electrode have been examined through XPS analysis, specifically focusing on the Li 1s and F 1s spectra, as depicted in Figure S7 (Supporting Information). Following 200 cycles (Figure 5j), the original morphology of Si_0.50_Ge_0.50_H vanishes entirely, and the micrograph illustrates the severe pulverization of electrode particles into smaller fragments. Concurrently, the thick SEI pieces coalesce and become denser, while significant Si_0.50_Ge_0.50_H restructuring results in a complete fusion with the surrounding SEI film. The SEM‐EDX elemental mapping (Figure S11, Supporting Information) reveals a homogeneous distribution of Si, Ge, and O atoms throughout the active substance after 10, 100 and 200 cycles when compared to the initial state (0 cycles). This observation demonstrates the absence of phase separation within the Si_0.50_Ge_0.50_H solid solution.^[^ ^80^ ^]^ On the other hand, the presence of oxygen in the samples may have occurred during the transfer process, as the substrates were exposed to air at various stages of the transfer, potentially leading to the spontaneous oxidation of amorphous Si_0.50_Ge_0.50_.

**Figure 6:**
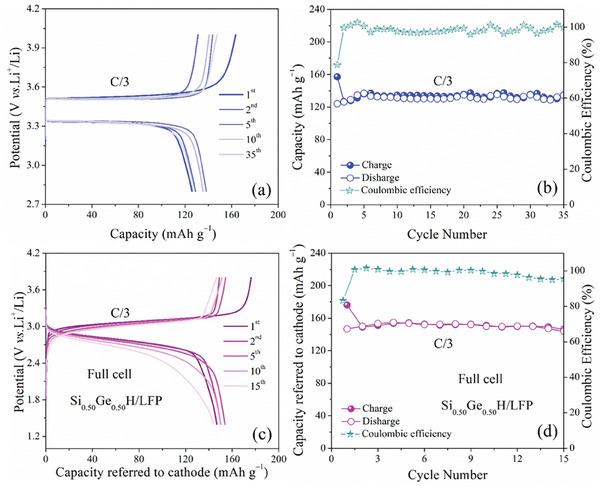
Selected voltage curves a,b) specific capacity and coulombic efficiency trends of LFP at a C/3 rate (1 C = 170 mA g^–1^) between 2.8 and 4.0 V. Electrochemical response of the Si_0.50_Ge_0.50_H/LFP full cell at a C/3 rate referred to the LFP cathode mass (1 C = 170 mA g^–1^) within the 1.4–3.8 V range in terms of selected voltage profiles c,d) specific capacity and coulombic efficiency vs. cycle number.

To assess the applicability of the Si_0.50_Ge_0.50_H anode material, lithium‐ion full cells were assembled, utilizing the mass of the cathode material (LFeiPO_4_, LFP) as a standard to measure the current density and specific capacity of the full cells. The electrochemical response of commercial LFP was investigated in a coin‐type half‐cell with a cycling test at a 0.33 C rate (1 C = 170 mA g^−1^). The half‐cell voltage profile (**Figure** **6**a) displays an irreversible plateau at about 3.5 V, revealing a stable charge capacity of 135 mAh g^−1^, as can be seen in Figure 6b.

Figure 6c,d depict the charge/discharge curves and cycling behavior of the Si_0.50_Ge_0.50_H/LFP full cell at a rate of 0.33 C (1 C = 170 mA g^−1^ referred to the cathode mass). The galvanostatic voltage profiles during the 1^st^, 2^nd^, 5^th^, 10^th^, and 15^th^ cycles exhibit a steady‐state signal at ≈2.9 V. Subsequently, the voltage values of the major sloped plateau shift to a minor one, possibly due to the irreversible rearrangement of the anode material during charge/discharge process.^[^ ^82^ ^]^ The first cycle displays a low Coulombic efficiency of ≈83.3%, which improves over subsequent cycles, reaching ≈99.2% after 10 cycles. This behavior is attributed to well‐known factors such as initial electrolyte decomposition, formation/stabilization of the SEI layer and structural reorganization of the anode, as reported in lithium half‐cells.^[^ ^83^ ^]^ As shown in Figure 6d, the full cell exhibits initial charge/discharge capacities of 153 mAh g_cathode_^−1^. After 15 cycles, a discharge capacity of 144 mAh g_cathode_^−1^ is retained with a high Coulombic efficiency (over 98.3%), indicating relatively favorable cycle stability of the Si_0.50_Ge_0.50_H/LFP full cell.

## 3 Conclusion

The electrode materials Si_0.25_Ge_0.75_H, Si_0.50_Ge_0.50_H and Si_0.75_Ge_0.25_H were synthesized through topotactic deintercalation of the Zintl‐phase precursor Ca(Si*_x_* Ge_1−_ *_x_*)_2_ crystals. These materials benefit from the simultaneous presence of both silicon, which provides high capacity at low cost, and germanium, which contributes to high‐rate performance and capacity retention. Among these compositions, the Si_0.50_Ge_0.50_H electrode reveals the best cycling performance, with a discharge capacity of 1059 mAh g^−1^ after 60 cycles at a current density of 75 mA g^−1^. To investigate the reaction mechanism, ex‐situ TEM, HRTEM and SAED techniques were employed, revealing the transformation of a mixture of amorphous and crystalline Si_0.50_Ge_0.50_H phases into *a*‐Si_0.50_Ge_0.50_ after the initial cycles, providing a comprehensive understanding of the dynamic behavior of the Si_0.50_Ge_0.50_H electrode during lithiation and delithiation processes. To gain further insight into the lithium storage behavior, ex‐situ Raman spectroscopy, XRD and SEM techniques were employed. The initial lithiation process resulted in the transformation of Si_0.50_Ge_0.50_H into a new *c*‐Li_15_(Si*_x_* Ge_1‐_ *_x_*)_4_ phase. Subsequently, lithiation occurred to form *a*‐Si_0.50_Ge_0.50_ phase. The Si_0.50_Ge_0.50_H electrode exhibits gradual capacity degradation in long‐term cycling, which can be attributed to relatively big volume changes, irreversible material agglomeration and structural evolution during electrochemical cycling. On the other hand, a deeper understanding of novel phases such as silicane‐germanane alloys will support their utilization as battery materials and offer alternative avenues to tackle the challenges associated with the lithiation of silicon‐based and germanium‐based materials.

## 4 Experimental Section

### Chemicals

Carbon black (CB, Super P, Alfa Aesar), poly(acrylic acid) (PAA, Sigma‐Aldrich), poly(vinylidene fluoride) (PVDF, Alfa Aesar), *N*‐methylpyrrolidone (NMP, 99.7%, Sigma‐Aldrich); and ethanol (C_2_H_5_OH, 99.8%, Sigma‐Aldrich) were purchased and used without any additional purification. Calcium (Ca, 99.9%), silicon (Si, 99.999%), and germanium (Ge, 99.999%) were acquired from Alfa Aesar, Germany. Hydrochloric acid (HCl, 37%) was bought from Penta, Czech Republic. The lithium hexafluorophosphate (LiPF_6_) solution mixed with ethylene carbonate (EC), dimethyl carbonate (DMC) and deiethyl carbonate (DEC) with the addition of vinylene carbonate (VC) was received from Sigma‐Aldrich.

### Synthesis of Zintl Phases

Zintl phases were synthesized using commercially available Si, Ge, and Ca, following a previously published procedure.^[^ ^17^ ^]^ To synthesize CaGe_1.5_Si_0.5_, a quartz glass ampoule with an aluminum oxide liner was utilized. A stoichiometric amount of Ca, Ge, and Si, corresponding to 10 g of CaGe_1.5_Si_0.5_, was carefully placed inside the ampoule. Subsequently, the ampoule was sealed under high vacuum conditions, maintaining a pressure of 1 × 10^−3^ Pa. The sealed ampoule containing the reaction mixture was then heated for 6 h at 1050 °C. After that, the ampoule was gradually cooled down to room temperature at a controlled rate of 0.5 °C min^−1^. Following the cooling process, the formed CaGe_1.5_Si_0.5_ crystals were mechanically separated from the ampoule. The synthesis of CaGe_1.0_Si_1.0_ and CaGe_0.5_Si_1.5_ followed a similar procedure, with the only variation being the adjustment of the elemental proportions to correspond to the desired compositions.

### Exfoliation of Zintl Phases

In the initial step, hydrochloric acid (100 mL) was cooled inside a Schlenk tube under an argon (Ar) atmosphere to –20 °C. One of the following compounds: CaGe_1.5_Si_0.5_ (1.0 g), CaGe_1.0_Si_1.0_ (1.0 g) or CaGe_0.5_Si_1.5_ (1.0 g) was added to the aqueous 37% HCl under the aforementioned Ar atmosphere. The resulting mixture was placed in a freezer at –20 °C for 10 days, during which it was shaken twice to facilitate the reaction. After completion of the reaction period, the exfoliated material was filtered and subsequently washed with cold HCl (100 mL), HF (5%, 100 mL), deionized water (3*×*100 mL) and methanol (2 *×*100 mL). The purified material was dried under vacuum to remove any remaining solvent. The resulting Si_0.25_Ge_0.75_H, Si_0.50_Ge_0.50_H and Si_0.75_Ge_0.25_H materials were then stored under an inert Ar atmosphere in a glovebox to prevent further oxidation.

### Computational Details

DFT calculations were performed using the Vienna Ab‐initio Simulation Package (VASP 5.4).^[^ ^84^, ^85^ ^]^ The generalized gradient approximation (GGA) in the scheme of Perdew‐Burke‐Ernzerhof (PBE) functional for the exchange‐correlation energy was used.^[^ ^86^ ^]^ A cutoff energy of 500 eV was set for the plane‐wave expansion of the electronic wave functions and the energy convergence criterion for electronic relaxation was set to 10^−5^ eV. The Brillouin zone integrations were performed by using a 5 × 5 × 1 Gamma‐centered *k*‐point mesh. To accurately evaluate the long‐range van der Waals interactions, the semiempirical correction method (DFT‐D3) was employed.^[^ ^87^ ^]^ A 20 Å vacuum region in the *z* direction was set for the periodic boundary condition. The method of climbing‐image nudged elastic band (CI‐NEB) was applied to examine the diffusion pathways and diffusion barriers.^[^ ^88^ ^]^

### Material Characterization

Thermal gravimetric mass spectrometry (TG‐MS) was carried out under an Ar atmosphere, with the temperature ranging from 25 to 650 °C and a heating rate of 5 °C min^−1^. This technique was utilized to detect gaseous decomposition products during the thermal treatment of the three materials. Raman spectroscopy was performed using a Renishaw in Via system (UK), utilizing a charge‐coupled device detector and a green DPSS laser with a wavelength of 532 nm and a power output of 50 mW. The measurements were performed at ambient conditions with a 20× objective, a 10 s integration time, and a laser power of 10 mW for each measurement. X‐ray diffraction (XRD) analysis was conducted on the samples (Si_0.25_Ge_0.75_H, Si_0.50_Ge_0.50_H and Si_0.75_Ge_0.25_H) using Cu Kα radiation in a Bruker D8 Advance diffractometer (Germany). The measurements covered a scattering angle (2θ) range of 5°−60° with a scanning step size of 0.02°. X‐ray photoelectron spectroscopy (XPS) was carried out using an Omicron Nanotechnology Ltd instrument (Germany) equipped with a monochromatic Al X‐ray radiation source (K_α1_ = 1486.7 eV). This technique was utilized to identify the chemical compositions and valence states of the elements. For morphological and structural analysis, a scanning electron microscope (SEM) Tescan MAIA 3 (Czech Republic) was used, while a transmission electron microscope (TEM) JEOL 2200 FS (Japan) was employed for detailed structural characterization. SEM‐energy‐dispersive X‐ray spectroscopy (SEM‐EDX) was performed via an Oxford Instruments system with a silicon drift detector X‐MaxN 80 TS to analyze the elemental composition. Fourier transform infrared spectroscopy (FTIR) measurements were conducted using an iS50R FTIR spectrometer (Thermo Scientific, USA) equipped with a DLaTGS detector and a KBr beam splitter. The spectral range analyzed was 4000–400 cm^–1^, with a resolution of 4 cm^–1^. The samples were prepared as KBr pellets containing 300 mg of KBr powder and 1.0 mg of sample.

### Battery Assembly and Electrochemical Measurements

The electrode fabrication were carried out inside an argon‐filled glovebox (MBRAUN, Germany) with O_2_ and H_2_O content below 0.1 ppm. The electrode slurry was prepared by mixing the active material (Si_0.25_Ge_0.75_H, Si_0.50_Ge_0.50_H and Si_0.75_Ge_0.25_H), PAA binder, and CB conducting agent in a weight ratio of 7:2:1 in NMP solvent. The consistent slurry was then coated onto a copper foil current collector with a thickness of 7 µm using a laboratory doctor blade. Subsequently, the coated slurry was heated on a ceramic heating plate (C‐MAG HS 7 digital) at 50 °C for 12 h to facilitate drying. The dried slurry was then cut into disks with a diameter of 10 mm, which served as the electrode. The mass loadings in the electrodes (Si_0.25_Ge_0.75_H, Si_0.50_Ge_0.50_H and Si_0.75_Ge_0.25_H) ranged from 0.9 to 1.2 mg cm^−2^. Two‐electrode half‐cells were assembled using CR2025 coin cells from MTI Corporation. Inside the glovebox, an electrode disk and a metallic lithium (Li) foil were combined, with a glass microfiber film (Whatman, Grade GF/D) separating them. These components were immersed in an electrolyte containing a solution of 1.0 m LiPF_6_ dissolved in a mixed solvent consisting of EC/DMC/DEC with a volume ratio of 1/1/1 (v/v/v), supplemented with 1.0% VC solvent.

Cyclic voltammetry (CV) and electrochemical impedance spectroscopy (EIS) measurements were carried out on a 2025‐type coin cell using an Autolab PGSTAT204 workstation (Eco Chemie, Utrecht, Netherlands). CV measurements were performed on the electrodes (Si_0.25_Ge_0.75_H, Si_0.50_Ge_0.50_H and Si_0.75_Ge_0.25_H) at a scanning rate of 0.2 mV s^−1^, covering a voltage range from 0.01 to 2.0 V vs. Li^+^/Li. Multiple CV tests were carried out at scanning rates ranging from 0.2, 0.4, 0.6, 0.8, 1.0 to 1.2 mV s^−1^. After 60 cycles, EIS tests were recorded under open circuit potential using an AC voltage (10 mV amplitude) in the 100 kHz to 100 mHz frequency range. When analyzing the obtained EIS data, the resulting spectra were processed using Nova 2.1 and subsequently fitted through ZView 2.

The cycling behavior and rate capability of the three electrodes were investigated in CR2025‐type coin cells. The cells were operated within a voltage range of 0.01 V to 2.0 V vs. Li^+^/Li. The cycling test involved 60 cycles, and each cycle was conducted at a current density of 75 mA g^−1^. Rate performance measurements were carried out at various current densities, specifically 75, 150, 300, 600, 900, and 1000 mA g^−1^. The long‐term cycling measurements comprised 1000 cycles, and each cycle was performed at current densities of 1000 and 2000 mA g^−1^, respectively.

To prepare the cathode electrode for lithium‐ion full cell, a mixture comprising 80 wt.% of active materials (LFP), 10 wt.% CB, and 10 wt.% PVDF was blended with NMP solvent. The resulting slurry was then coated onto aluminum foil, followed by drying at 60 °C in a vacuum oven and subsequently cutting into disks with a 10 mm diameter. In the configuration of full cells, Si_0.50_Ge_0.50_H and LFP, featuring a specific capacity ratio of 1: (1.9–2.3), were employed as anode and cathode materials, respectively. It is important to note that the current density settings and specific capacity calculations are based on the mass of LFP cathode material in the full cells. All galvanostatic cycling measurements were conducted at room temperature, using a Neware battery test system (BTX 7.6, Shenzhen, China).

### Battery Disassembly and Ex‐Situ Measurements

Each of the assembled coin cells underwent specific cycling, and after that, they were disassembled within an argon‐filled glovebox. All the recovered electrodes were cleansed with a DMC solvent to eliminate any remaining surface residues. Several ex‐situ characterization techniques were employed, including XRD, Raman spectroscopy, SEM, TEM, HRTEM, and SAED.

## Conflict of Interest

The authors declare no conflict of interest.

## Supporting information

Supporting Information

## Data Availability

The datasets generated during and/or analyzed during the study are accessible via the Zenodo repository: 10.5281/zenodo.10951039.
