# Supplementary material for: Reaction Mechanism and Performance of Innovative 2D Germanane‐Silicane Alloys: Si x Ge1− x H Electrodes in Lithium‐Ion Batteries
Source: Adv Sci (Weinh). 2024 Apr 22;11(24):2308955. doi: 10.1002/advs.202308955 (PMC11199986; doi:10.1002/advs.202308955)
Supplement: Supplementary file 1 — Supporting Information [file ADVS-11-2308955-s001.pdf]

ADVANCED SCIENCE
Open Access

Supporting Information

for *Adv. Sci.*, DOI 10.1002/advs.202308955

Reaction Mechanism and Performance of Innovative 2D Germanane-Silicane Alloys: $Si_xGe_{1-x}H$ Electrodes in Lithium-Ion Batteries

Shuangying Wei, Tomáš Hartman, Stefanos Mourdikoudis, Xueting Liu, Gang Wang, Evgeniya Kovalska, Bing Wu, Jalal Azadmanjiri, Ruizhi Yu, Levna Chacko, Lukas Dekanovsky, Filipa M. Oliveira, Min Li, Jan Luxa, Saeed Jamali Ashtiani, Jincang Su* and Zdeněk Sofer**

Supporting information

Reaction Mechanism and Performance of Innovative 2D Germanane-Silicane Alloys: $Si_xGe_{1-x}H$ Electrodes in Lithium-Ion Batteries

Shuangying Wei[1*], Tomáš Hartman[1], Stefanos Mourdikoudis[1], Xueting Liu[2], Gang Wang[2], Evgeniya Kovalska[3], Bing Wu[1], Jalal Azadmanjiri[1], Ruizhi Yu[4], Levna Chacko[1], Lukas Dekanovsky[1], Filipa M Oliveira[1], Min Li[1,5], Jan Luxa[1], Saeed Jamali Ashtiani[1,6], Jincang Su[2*], Zdeněk Sofer[1*]

[1] *Department of Inorganic Chemistry, University of Chemistry and Technology Prague, Technická 5, Prague 6, 16628, Czech Republic*

[2] *School of Materials Science and Engineering, Xiangtan University, Xiangtan 411105, China*

[3] *Department of Engineering, Faculty of Environment, Science and Economy, University of Exeter, Exeter, EX4 4PY, United Kingdom*

[4] *Institute of Micro/Nano Materials and Devices, Ningbo University of Technology, Ningbo, 315211 China*

[5] *School of Physics, Xi'an Jiaotong University, Xi'an 710049, China*

[6] *Department of Physical Chemistry, University of Chemistry and Technology Prague, Technická 5, Prague 6, 16628, Czech Republic*

Corresponding authors: weis@vscht.cz; sujc@xtu.edu.cn; zdenek.sofer@vscht.cz

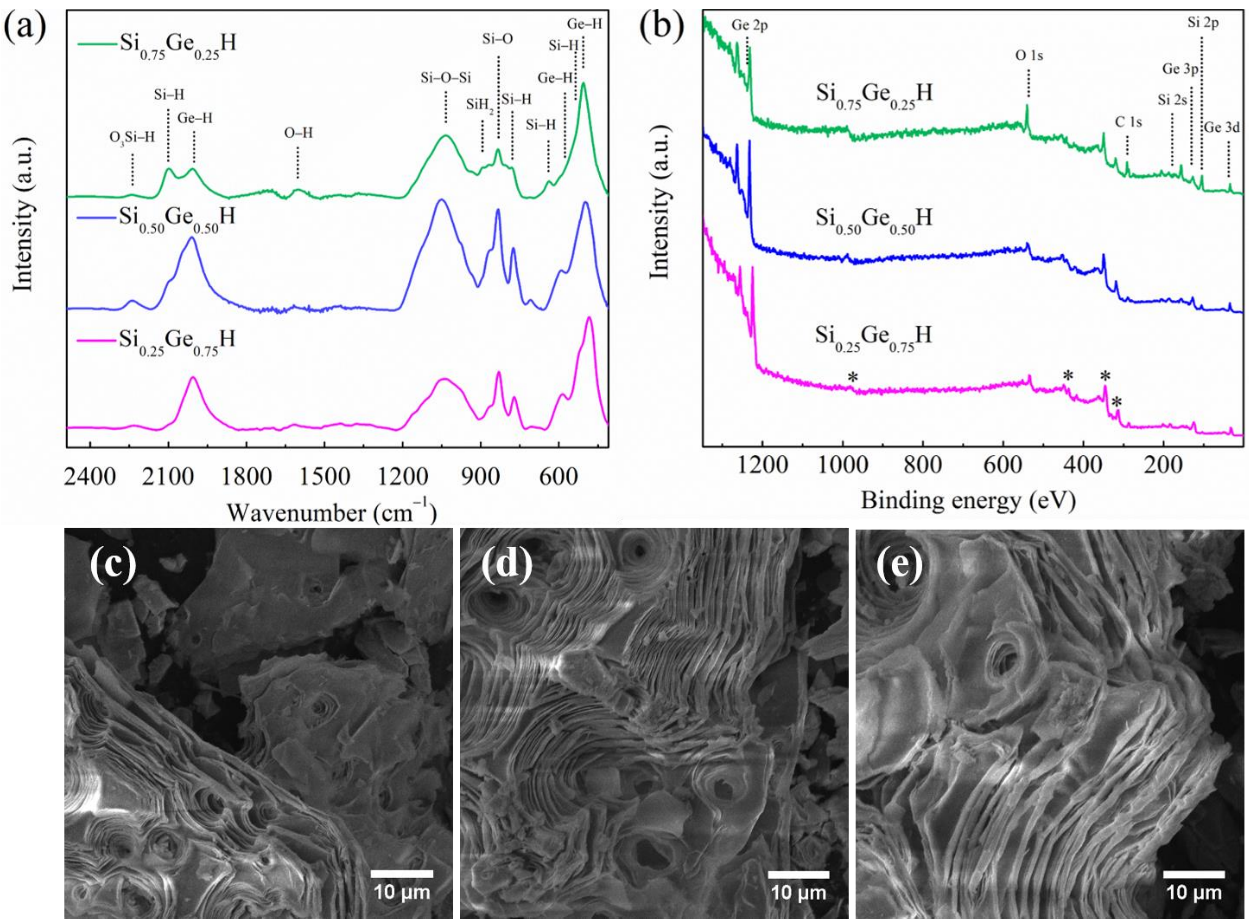

Fig. S1 FTIR spectra **a** and XPS survey spectra **b** of $Si_{0.25}Ge_{0.75}H$, $Si_{0.50}Ge_{0.50}H$ and $Si_{0.75}Ge_{0.25}H$; SEM images of $Si_{0.25}Ge_{0.75}H$ **c**, $Si_{0.50}Ge_{0.50}H$ **d** and $Si_{0.75}Ge_{0.25}H$ **e**.

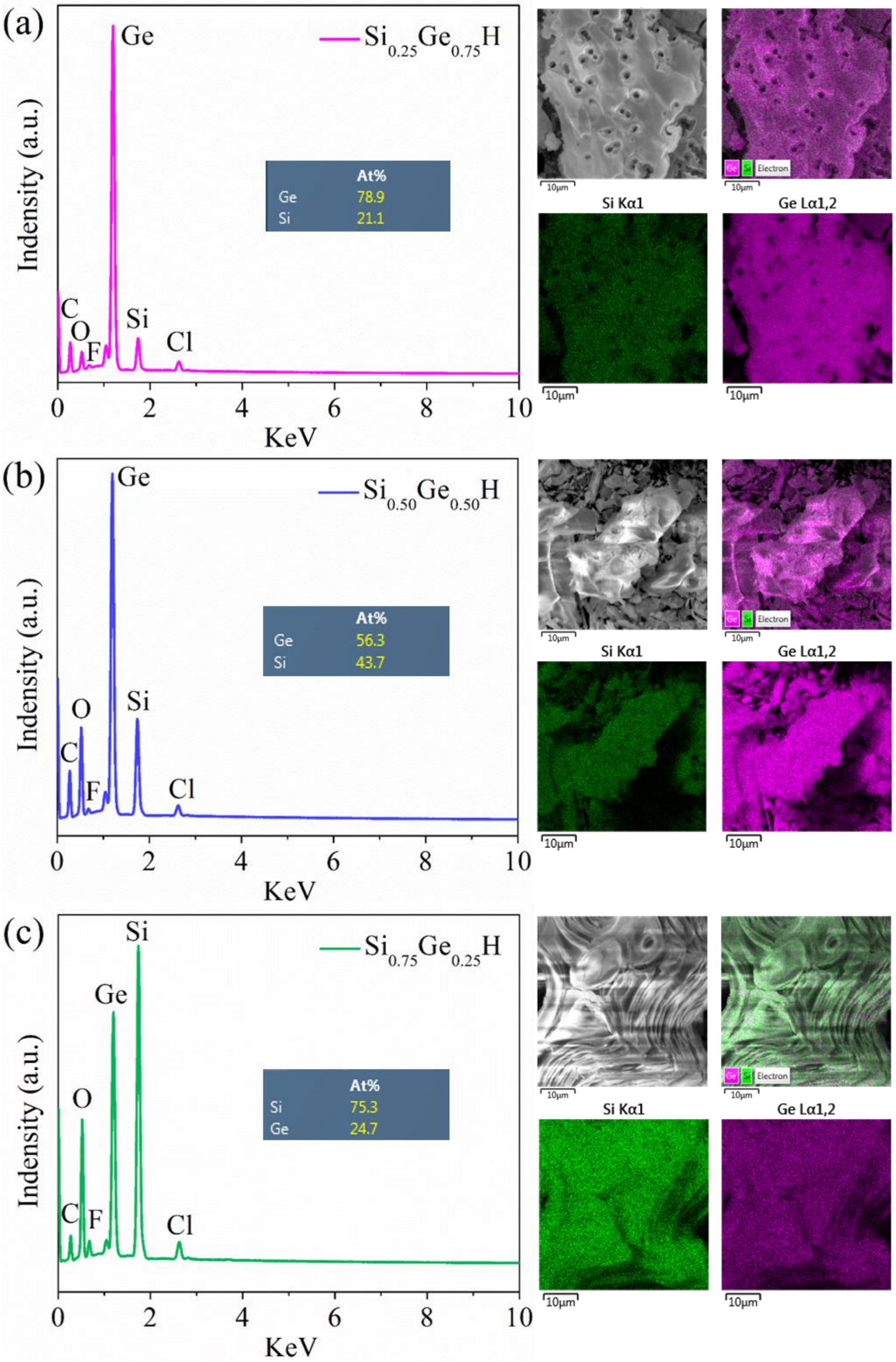

Fig. S2 Mapping of elements and respective EDX spectra of $Si_{0.25}Ge_{0.75}H$ **a**, $Si_{0.50}Ge_{0.50}H$ **b** and $Si_{0.75}Ge_{0.25}H$ **c**.

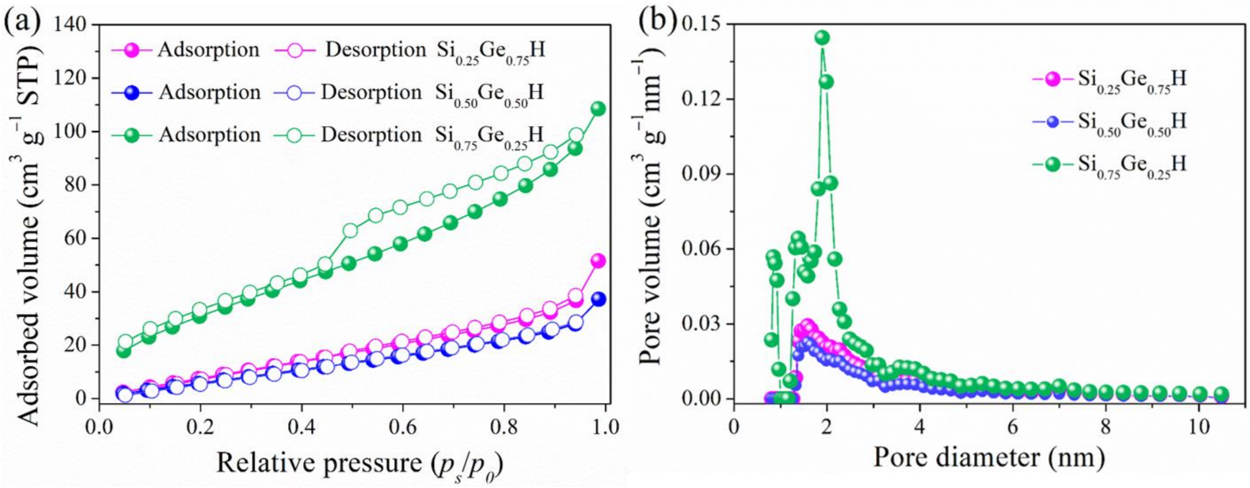

Fig. S3 Nitrogen adsorption–desorption isotherms **a** and pore diameter distributions calculated from N_2 desorption isothermals **b** for $Si_{0.25}Ge_{0.75}H$, $Si_{0.50}Ge_{0.50}H$ and $Si_{0.75}Ge_{0.25}H$, respectively.

Table 1. Specific surface area and total pore volume of $Si_{0.25}Ge_{0.75}H$, $Si_{0.50}Ge_{0.50}H$ and $Si_{0.75}Ge_{0.25}H$.

Samples	BET surface area ($m^2\ g^{-1}$)	Total pore volume ($cm^3\ g^{-1}$)
$Si_{0.25}Ge_{0.75}H$	21.26	0.0562
$Si_{0.50}Ge_{0.50}H$	16.14	0.0418
$Si_{0.75}Ge_{0.25}H$	81.99	0.1434

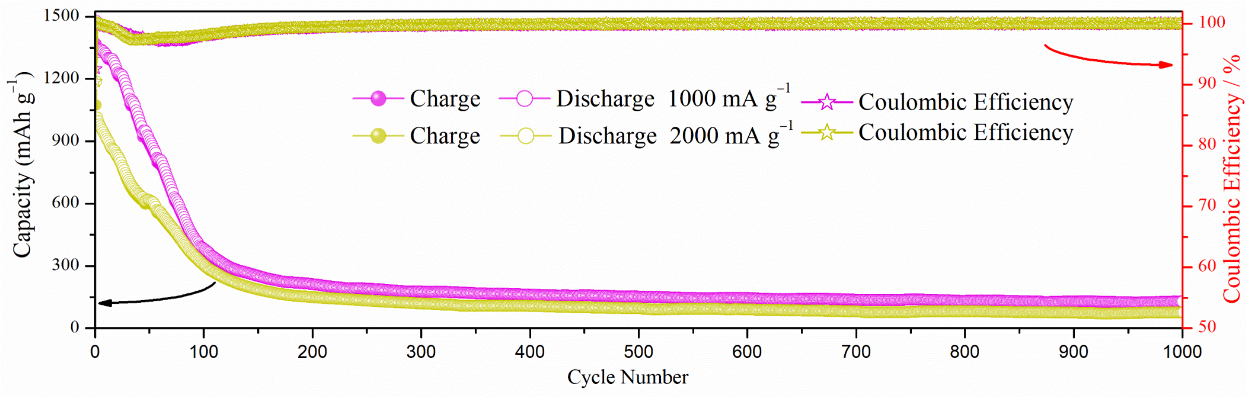

Fig. S4 Cycling stability of $Si_{0.50}Ge_{0.50}H$ electrode at current densities of 1000 and 2000 mA g^{-1} over 1000 cycles.

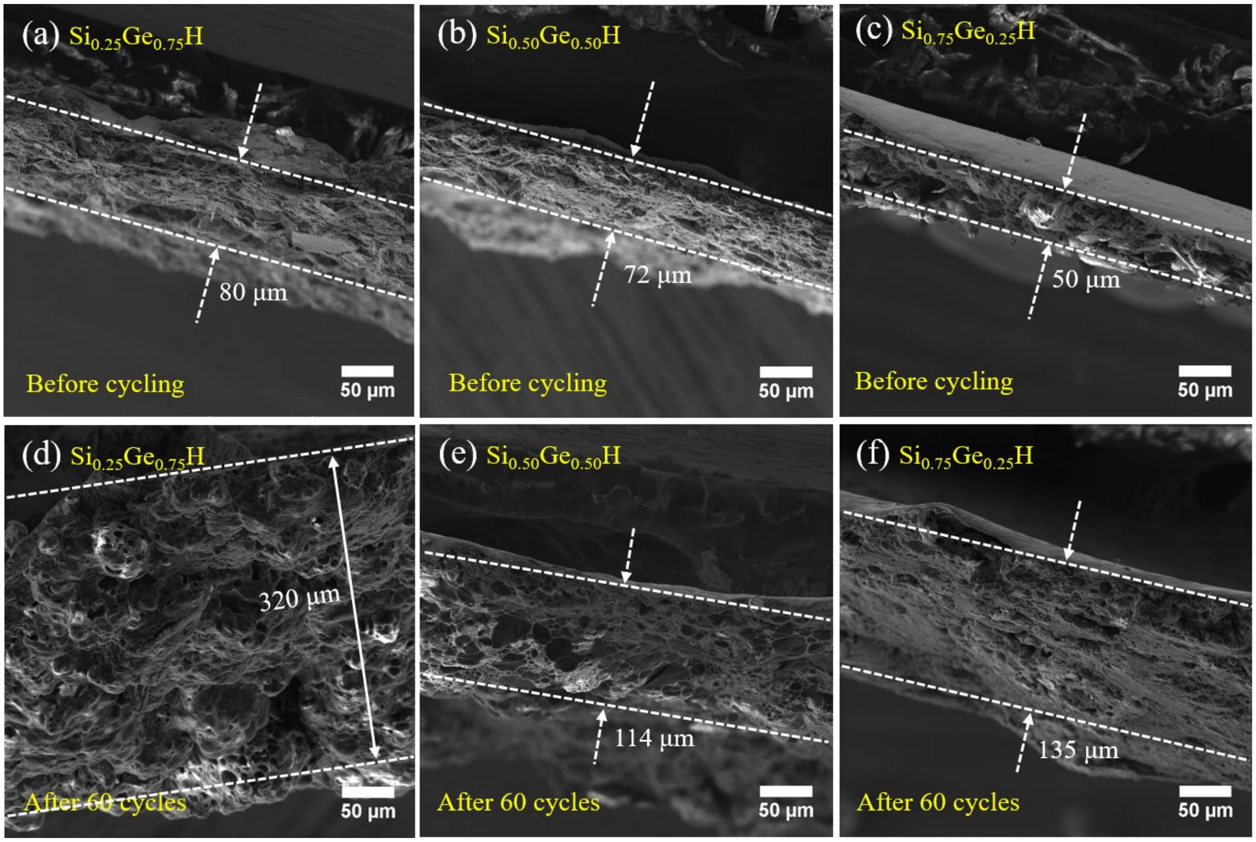

Fig. S5 Cross-section SEM images illustrating thickness deformation evolution in $Si_{0.25}Ge_{0.75}H$ (**a**, **d**), $Si_{0.50}Ge_{0.50}H$ (**b**, **e**) and $Si_{0.75}Ge_{0.25}H$ (**c**, **f**) electrodes before and after 60 cycles, respectively.

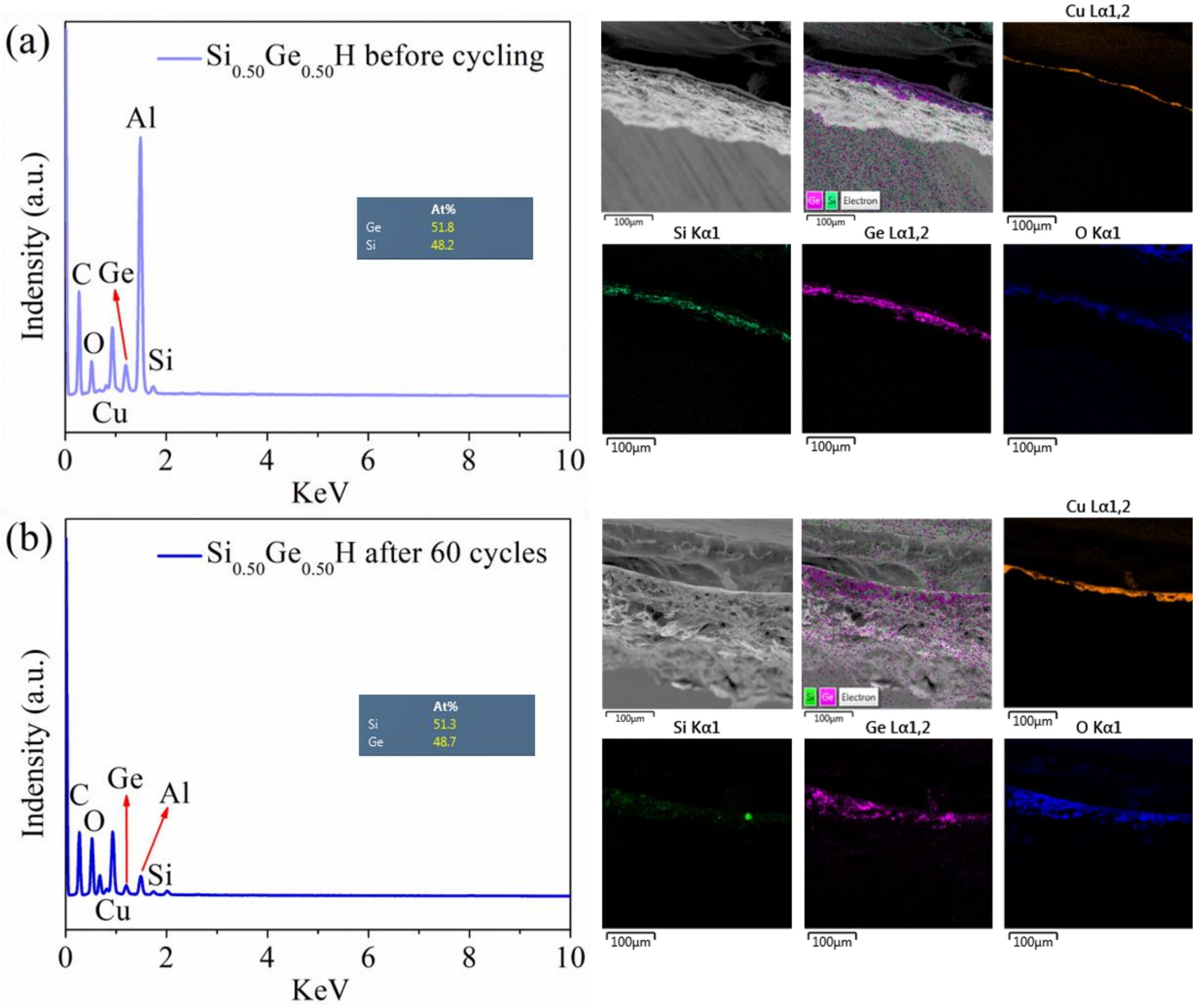

Fig. S6 Mapping of elements and corresponding EDX spectra of $Si_{0.50}Ge_{0.50}H$ electrode before and after 60 cycles (**a**, **b**), respectively.

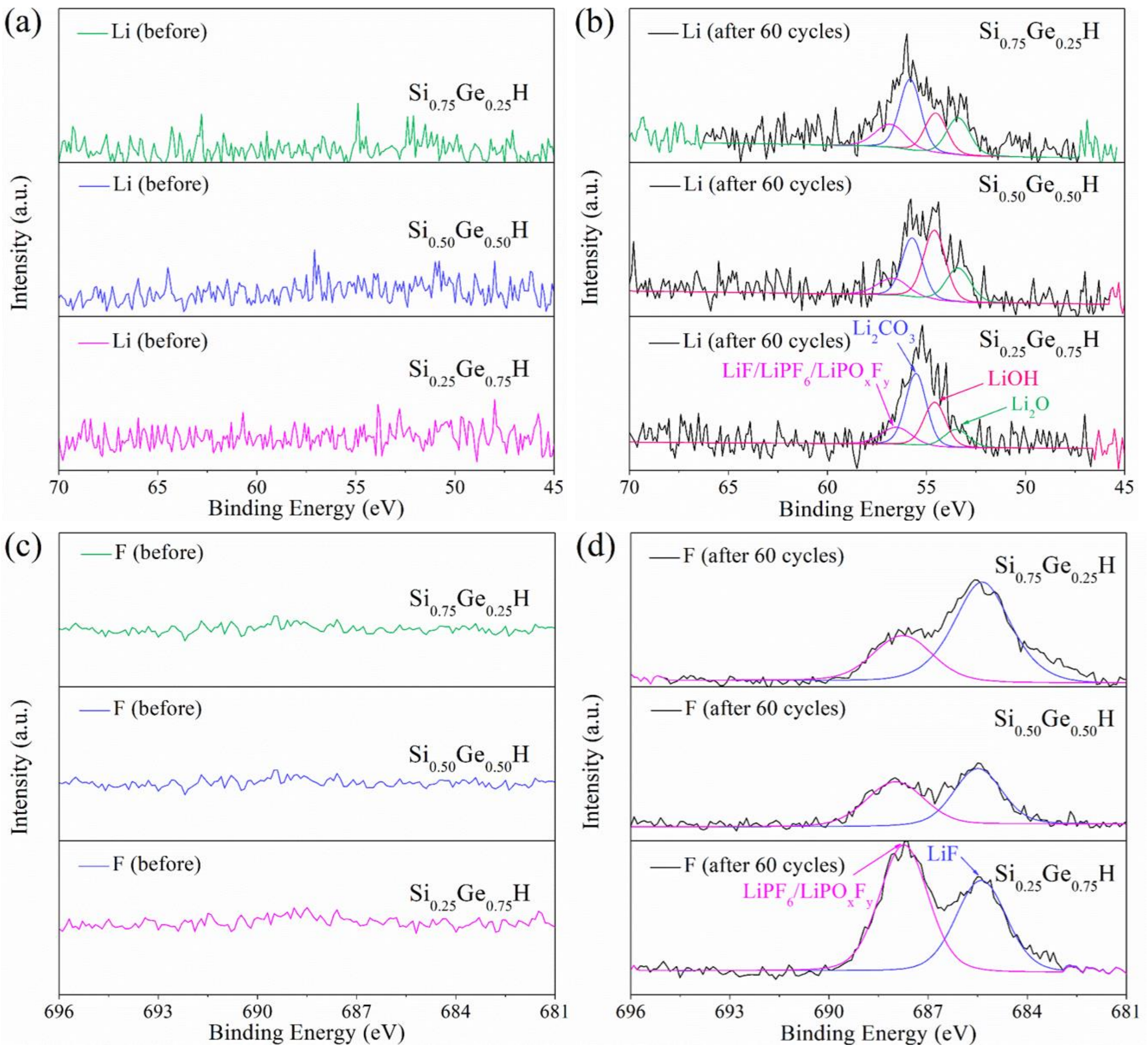

Fig. S7 XPS spectra of **a**, **b** Li 1*s*, and **c**, **d** F 1*s* core levels for $Si_{0.25}Ge_{0.75}H$, $Si_{0.50}Ge_{0.50}H$ and $Si_{0.75}Ge_{0.25}H$ before **a**, **c** and after 60 cycles **b**, **d**, respectively.

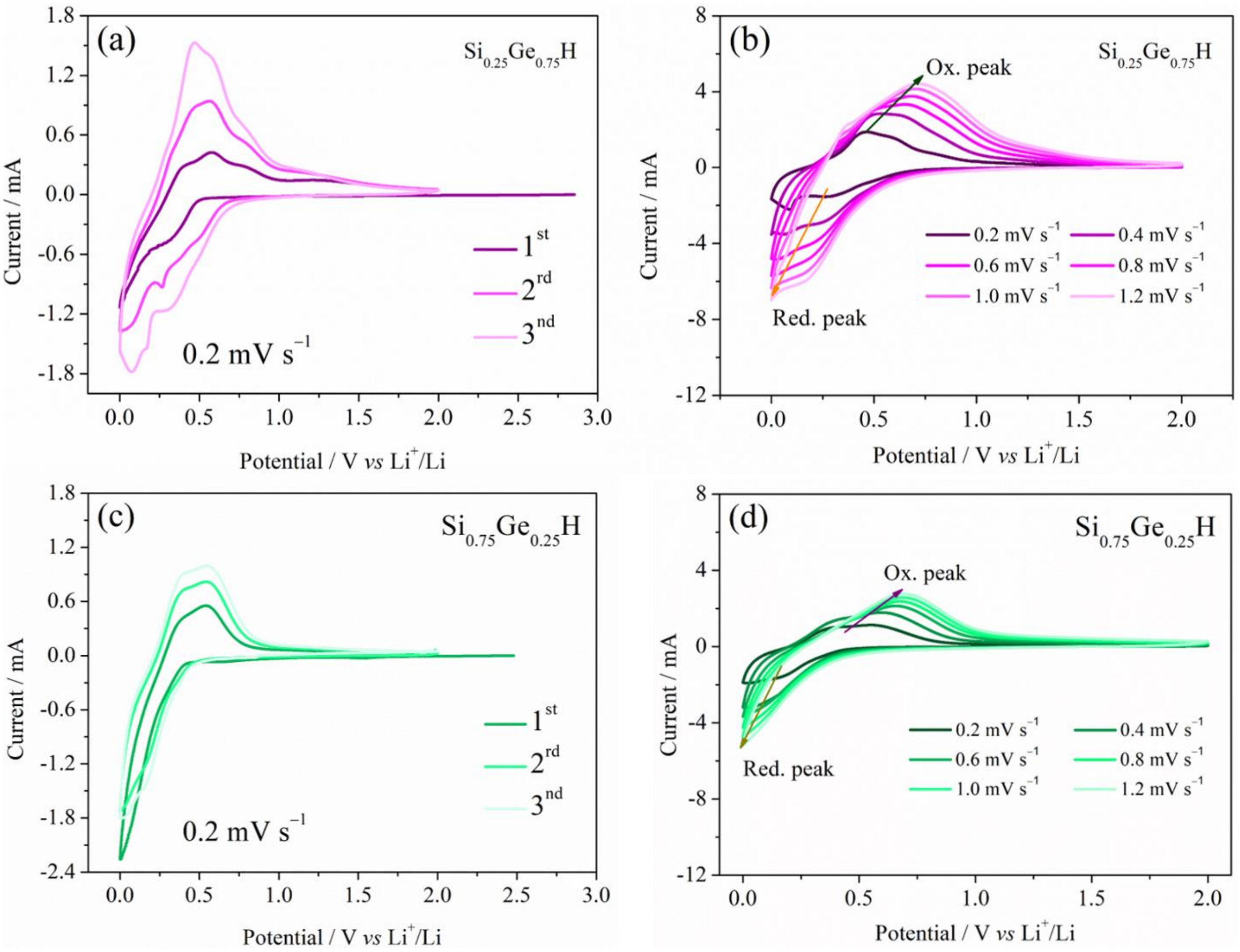

Fig. S8 **a** and **c** an initial 3-cycle CV curves of $Si_{0.25}Ge_{0.75}H$ and $Si_{0.75}Ge_{0.25}H$ electrodes at 0.2 mV s^{-1}; **b** and **d** CV of $Si_{0.25}Ge_{0.75}H$ and $Si_{0.75}Ge_{0.25}H$ electrodes at 0.2 – 1.2 mV s^{-1}.

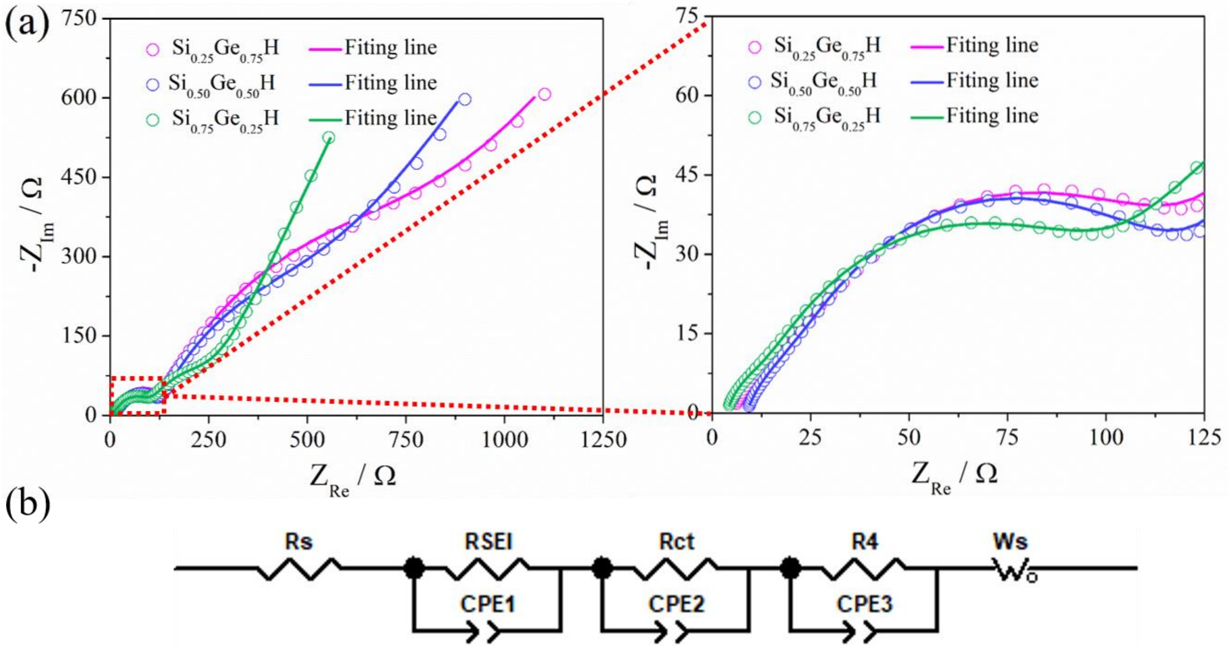

Fig. S9 Nyquist plots of $Si_{0.25}Ge_{0.75}H$, $Si_{0.50}Ge_{0.50}H$, and $Si_{0.75}Ge_{0.25}H$ electrodes after 60 cycles **a** and the corresponding equivalent circuit model **b**.

Table S2. Impedance parameters derived using equivalent circuit model (Figure S9b) for $Si_{0.25}Ge_{0.75}H$, $Si_{0.50}Ge_{0.50}H$, and $Si_{0.75}Ge_{0.25}H$ after 60 cycles under open circuit potential condition, In detail: equivalent circuit, electrode/electrolyte interface resistances (R_i, *i* = s, SEI, ct, 4) and χ^2 parameter.

Electrodes	Equivalent circuit	R_s (Ω)	R_{SEI} (Ω)	R_{ct} (Ω)	R_4 (Ω)	χ^2
$Si_{0.25}Ge_{0.75}H$	$R_s(R_{SEI}CPE_1)(R_{ct}CPE_2)(R_4CPE_3)W_s$	5.190	95.07	15.07	585.5	2.0×10^{-4}
$Si_{0.50}Ge_{0.50}H$	$R_s(R_{SEI}CPE_1)(R_{ct}CPE_2)(R_4CPE_3)W_s$	8.691	93.08	12.05	295.9	1.5×10^{-4}
$Si_{0.75}Ge_{0.25}H$	$R_s(R_{SEI}CPE_1)(R_{ct}CPE_2)(R_4CPE_3)W_s$	3.726	90.31	7.891	135.3	1.1×10^{-4}

Table S3 Recently reported electrochemical performance of SiGe-based anode materials for LIBs.

Material structure	Specific capacity (mAh g^{-1})	Current density (mA g^{-1})	Cycling number	Battery type
$Si_{0.5}Ge_{0.5}$	1142	50	1	LIBs [1]
$Ge_{0.1}Si_{0.9}$	1020	C/2	100	LIBs [2]
$Si_{0.67}Ge_{0.33}$	1360	C/5	250	LIBs [3]
Si−Ge heterostructure nanowires	1180	C/5	400	LIBs [4]
This work ($Si_{0.50}Ge_{0.50}H$)	1059	75	60	LIBs

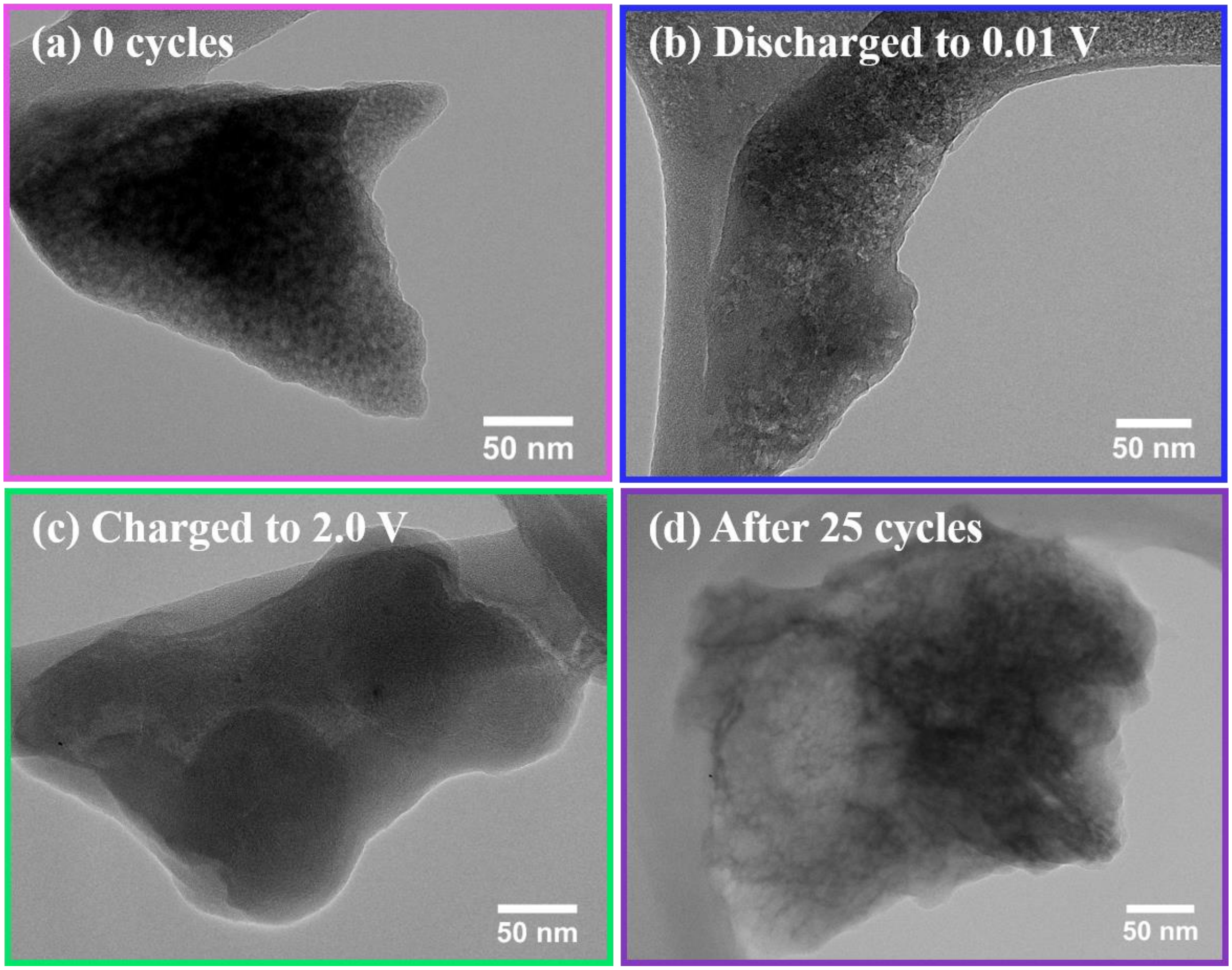

Fig. S10 TEM images depicting the evolution of morphological deformation in the $Si_{0.50}Ge_{0.50}H$ electrode during lithiation and delithiation: 0 cycles **a**, discharged to 0.01 V **b**, charged to 2.0 V **c** and after 25 cycles **d**.

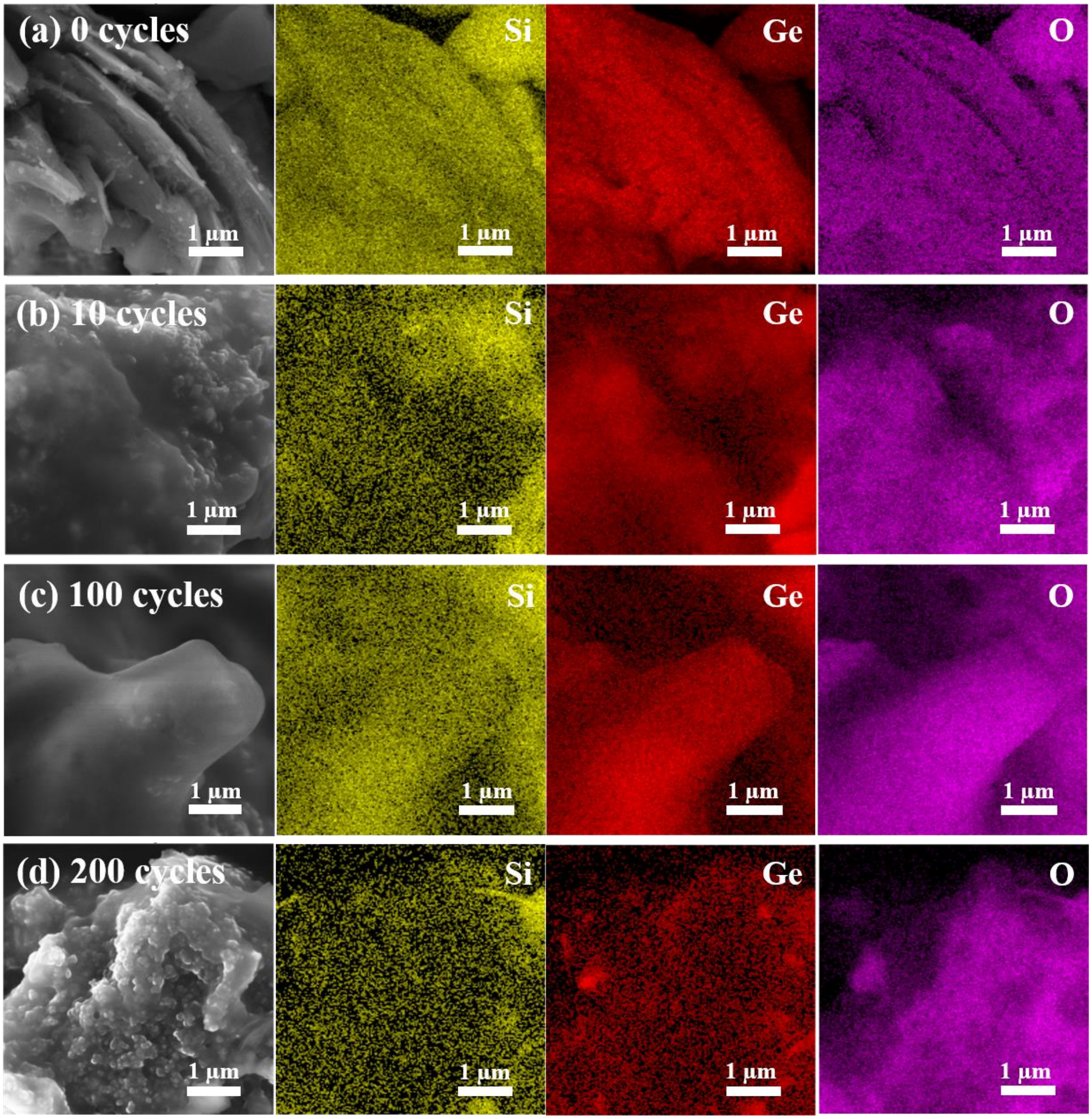

Fig. S11 SEM-EDX images illustrating morphological changes in the $Si_{0.50}Ge_{0.50}H$ electrode during lithiation and delithiation: 0 cycles **a**, after 10 cycles **b**, 100 cycles **c** and 200 cycles **d**.

References

[1] X. Chen, L. C. Loaiza, L. Monconduit, V. Seznec, *ACS Appl. Energy Mater.* **2021**, *4*, 12552.

[2] D. Duveau, B. Fraisse, F. Cunin, L. Monconduit, *Chem. Mater.* **2015**, *27*, 3226.

[3] K. Stokes, H. Geaney, G. Flynn, M. Sheehan, T. Kennedy, K. M. Ryan, *ACS Nano* **2017**, *11*, 10088.

[4] K. Stokes, G. Flynn, H. Geaney, G. Bree, K. M. Ryan, *Nano Lett.* **2018**, *18*, 5569.
